# Eating disorders, disordered eating, and body image research in New Zealand: a scoping review

**DOI:** 10.1186/s40337-022-00728-1

**Published:** 2023-01-17

**Authors:** Lana Cleland, Hannah L. Kennedy, Michaela A. Pettie, Martin A. Kennedy, Cynthia M. Bulik, Jennifer Jordan

**Affiliations:** 1 Department of Psychological Medicine, University of Otago, PO Box 4345, Christchurch, 8140 New Zealand; 2 Department of Pathology and Biomedical Science, University of Otago, Christchurch, New Zealand; 3 Department of Psychiatry, University of North Carolina at Chapel Hill, Chapel Hill, USA; 4 Department of Nutrition, University of North Carolina at Chapel Hill, Chapel Hill, USA; 5 Department Medical Epidemiology and Biostatistics, Karolinska Institutet, Stockholm, Sweden; 6 Mental Health Clinical Research Unit, Te Whatu Ora, Waitaha, Christchurch, New Zealand

**Keywords:** Eating disorders, Anorexia nervosa, Bulimia nervosa, Binge eating disorder, Scoping review, New Zealand

## Abstract

**Background:**

The prevention and treatment of eating disorders relies on an extensive body of research that includes various foci and methodologies. This scoping review identified relevant studies of eating disorders, body image, and disordered eating with New Zealand samples; charted the methodologies, sample characteristics, and findings reported; and identified several gaps that should be addressed by further research.

**Methods:**

Using scoping review methodology, two databases were searched for studies examining eating disorders, disordered eating, or body image with New Zealand samples. Snowball methods were further used to identify additional relevant articles that did not appear in initial searches. Two independent reviewers screened the titles and abstracts of 473 records. Full text assessment of the remaining 251 records resulted in 148 peer-reviewed articles being identified as eligible for the final review. A search of institutional databases yielded 106 Masters and Doctoral theses for assessment, with a total of 47 theses being identified as eligible for the final review. The included studies were classified by methodology, and the extracted information included the study foci, data collected, sample size, demographic information, and key findings.

**Results:**

The eligible studies examined a variety of eating disorder categories including binge-eating disorder, bulimia nervosa, and anorexia nervosa, in addition to disordered eating behaviours and body image in nonclinical or community samples. Methodologies included treatment trials, secondary analysis of existing datasets, non-treatment experimental interventions, cross-sectional observation, case-control studies, qualitative and mixed-methods studies, and case studies or series. Across all of the studies, questionnaire and interview data were most commonly utilised. A wide range of sample sizes were evident, and studies often reported all-female or mostly-female participants, with minimal inclusion of males and gender minorities. There was also an underrepresentation of minority ethnicities in many studies, highlighting the need for future research to increase diversity within samples.

**Conclusion:**

This study provides a comprehensive and detailed overview of research into eating disorders and body image in New Zealand, while highlighting important considerations for both local and international research.

## Plain English summary

Research into eating disorders should include different methods, and should be relevant to people of different ages, gender identities, and ethnicities. We completed a scoping review of research into eating disorders, disordered eating, and body image in New Zealand samples. We searched academic databases for relevant articles, and then screened the articles for eligibility. We then hand-searched key articles, and searched databases again using the names of key authors. A total of 148 peer reviewed articles and 47 theses were eligible for the review, and from these we extracted data on the study method, sample characteristics, and the focus and results. A wide range of methods and sample sizes were reported, and the studies explored several different eating disorders, as well as disordered eating and body image in nonclinical samples. However, the studies often involved all or mostly female samples, few to no gender minority participants, and an underrepresentation of minority ethnicities. Funders should provide adequate time and financial resources to fund recruitment from historically under-represented groups, emphasising their involvement as active researchers. In addition, funders should consider financing the use of novel or underutilised methods to advance knowledge in this field.

## Introduction

Eating disorders such as binge-eating disorder (BED), bulimia nervosa (BN), and anorexia nervosa (AN) are complex and potentially life-threatening psychiatric illnesses. Research in the New Zealand population suggests a lifetime prevalence of 1.9% for BED, 1–1.3% for BN, and 0.6% for AN [1, 2]. These disorders create a significant burden upon the lives of those affected, with many individuals facing prolonged periods of inpatient treatment or multiple relapses. Although research into eating disorders has made substantial progress in recent years, the limited success of available treatments underscores the need for a more complete picture of how to best understand and approach this cluster of disorders.

In addition to the more commonly acknowledged eating disorders noted above, there is a growing awareness surrounding those whose symptoms fall within the Diagnostic and Statistical Manual (DSM-5) [3] other specified feeding and eating disorders (OSFED) diagnostic category. These disorders include atypical or subthreshold forms of BN, AN, and purging disorder which previously were included in the DSM-IV eating disorder not otherwise specified (EDNOS) category, and the newly included night eating syndrome. Despite this group of disorders having been identified as being the most prevalent [4], research surrounding them is comparatively sparse.

At a sub-threshold level, eating disorder psychopathology is common in New Zealand, and has been reported in adolescents, university students, and middle-aged samples [5–7]. Disordered eating is often tightly intertwined with body dissatisfaction—a core symptom in the diagnostic criteria for AN and BN [3], which is also suggested to be relevant for BED [8]. Body dissatisfaction is regarded as a significant risk factor for the development of eating disorders [9, 10], with etiological models commonly citing the relationship between body dissatisfaction and subthreshold disordered eating. Body dissatisfaction can be seen as almost normative among young women and, increasingly, young men [11]. In light of this, our understanding of disordered eating can be supplemented by research into body dissatisfaction at both a clinical and subthreshold level.

Although many aspects of eating disorders, subthreshold disordered eating, and body dissatisfaction are studied extensively internationally, it is often unclear whether findings generalise to a New Zealand population. Moreover, even where such findings are applicable, there remains a need to understand these issues in a manner consistent with New Zealand’s unique sociocultural context [12, 13]. Achieving this requires a comprehensive body of research to be conducted within New Zealand, ideally with a range of study designs to ensure a detailed and broad understanding of these issues. Moreover, this research should adequately cover the range of issues pertaining to body image and eating disorders, and include samples that are representative of the population as a whole (such as Indigenous Māori and Pasifika populations). To this end, it is critical that local researchers are aware of what is available within the literature and what is lacking, thus informing the direction for future research and methodologies. However, we were unable to identify any comprehensive reviews of relevant studies involving New Zealand-based participants, thereby hindering progression of research into the issues at hand.

In an effort to bridge the gap between extant research and future projects, the present review scopes and synthesises the foci reported by studies examining eating disorders, disordered eating, and body image within studies that include New Zealand samples. This review was informed by scoping methodology outlined by the Preferred Reporting Items for Systematic Review and Meta-Analysis extension for Scoping Review (PRISMA-ScR) [14]. It involved: (a) the identification of relevant journal articles and theses; (b) charting the foci, methodologies, sample characteristics, and findings reported in the identified literature; and (c) a descriptive review of what was included, as well as gaps and areas which may be expanded upon.

## Methods

### Research question

The scoping review was informed by the research question: “To date, what are the methodologies and results reported by studies that have examined eating disorders, disordered eating, and body image in clinical and non-clinical samples in New Zealand?”.

### Eligibility

Meeting initial eligibility criteria was dependent on (1) the full text being available, (2) some portion of the sample living in New Zealand during the research, (3) the article or thesis being available in English, (4) the record not being a duplicate, and (5) the topic or a part of the focus being within scope. The scope was informed by the overarching research question of this review, and research items needed to include an examination of eating disorders, disordered eating, or body image in New Zealand samples.

Included eating disorder diagnoses were BED, BN, and AN in addition to disorders in the Other Specified Feeding and Eating Disorder (OSFED) category (DSM-5) or the former Eating Disorder Not Otherwise Specified (EDNOS) category (DSM-IV-TR) [15]. Also included were studies where only symptoms of these disorders (e.g. binge eating, purging) were assessed. Not included were Avoidant/Restrictive Food Intake Disorder (ARFID), pica and rumination disorder; categories shifted to the eating disorders section of DSM-5 from the DSM-IV-TR Feeding and Eating Disorders of Early Childhood Section [3, 15]. Body image in the context of this review included perceptions of one’s own body shape and size, but excluded research items that focused only on concerns such as perceived facial flaws [16], which are often a feature of body dysmorphic disorder. Lastly, research on samples of clinicians working in eating disorder treatment were included, given that this adds considerably to knowledge surrounding eating disorders and their treatment in New Zealand.

Both qualitative and quantitative studies were deemed in scope, as were case studies and case series. International studies that included original data from one or more New Zealand participants were included; however, meta analyses and systematic reviews were not, given that relevant data were likely already published elsewhere. It was decided that conference abstracts would be excluded, given that the findings were either published elsewhere, or the abstracts did not include sufficient information to meet basic eligibility criteria. Lastly, any trials that were in progress but unpublished were also excluded, as it would not be possible to chart the findings of those studies.

### Initial database search

To locate references for journal articles from a wide range of sources, relevant search terms were entered into Ovid (EMBASE, psychINFO). The search terms “eating disorder*.kw”, “anorexia nervosa.kw”, “bulimia nervosa.kw”, “binge eating disorder.kw”, “disordered eating.kw”, and “body image.kw” were combined using the “OR” function. This result was then combined with “new zealand.af” using the AND function, and the results were deduplicated. No additional search limitations were used in Ovid. The cut-off date for this and subsequent searches was set to 20 May, 2021.

### Snowball searches

During the initial screen of records returned in Ovid, seven authors known to publish research within this scope frequently appeared as first authors. Publications from these authors were further searched in Ovid by entering the search terms “jordan jennifer.au”, “carter frances a.au”, “gendall kelly a.au”, “mcintosh virginia v w or mcintosh virginia violet williams or mcintosh virginia vw).au”, “bulik cynthia m.au”, “wilksch simon m or wilksch sm.au”, “latner janet d or latner jd.au”. These searches were combined using the OR function, and the result was then combined with “new zealand.af” using the AND function. The results were deduplicated within Ovid before being merged with the initial OVID search records, and the combined results were again deduplicated.

The citations within key papers were also hand-searched by two reviewers (HK and LC) for additional relevant publications within New Zealand. Key papers included relevant epidemiological studies and treatment trials known among New Zealand eating disorders researchers. Referenced papers were then located and screened using the same criteria and checklist. Furthermore, when papers reporting secondary analyses referred back to publications which described original study samples, those publications were identified and screened for inclusion.

### Grey literature search

To locate Master’s and Doctoral theses, institutional research archives were searched for each of the University of Otago (OURArchive), University of Waikato (Research Commons), University of Canterbury (College of Science, College of Arts), Massey University (Massey Research Online), Auckland University of Technology (Open Repository), and Victoria University of Wellington (Open Access), and University of Auckland (ResearchSpace). A total of 29 potentially relevant theses, including 25 from the University of Auckland, were unavailable online or were only accessible only to staff and students at the relevant institutions. As such, full-text screening was unable to be completed for these records.

The terms “binge eating disorder”, “bulimia nervosa”, “anorexia nervosa”, and “body image” were entered into each university research archive and limited to thesis where possible. The terms “eating disorder” and “disordered eating” were also entered into the same archives. In some instances, these latter terms returned the same results as one of the initial four search terms, such as the results for “eating disorder” being the same as those for “binge eating disorder” in one database. In such cases, results were not added to the final number of records to be screened. In addition, when a very large number of unrelated results were returned for thesis search terms, the results for those terms were limited to “title contains”.

In some cases, the findings from grey literature had already been published in peer reviewed journals. To avoid overlap in these situations, the grey literature record was removed as a duplicate in favour of the published article. Further journal articles identified during this process were labelled as being found via snowball search.

### Record screening and eligibility

Search results from OVID were exported into EndNote, and then entered into an Excel spreadsheet to be screened separately by two blind reviewers (HK and LC). The reviewers first pre-screened the titles and abstracts of each record for relevance. Journal articles that were eligible for full-text searching were then located where possible, and the reviewers filled out a checklist to determine whether predetermined eligibility criteria were met. Following blind review, authors HK and LC met to discuss a small number of cases where the decision to include or exclude a record was inconsistent. In these cases, the records were further assessed and a final decision was agreed upon for each, with a total of 10 papers being discussed and 7 of these being excluded from the review.

### Data extraction and study classification

For each included research item, a range of data were extracted. The relevant population(s) or construct(s) of interest were identified, including any specific eating disorders being examined, disordered eating among nonclinical (NC) populations, or clinicians working within eating disorder treatment settings. The focus of each study was also briefly summarised, as were the key data collection instruments or measures. Gender and ages of participants were recorded as specified in the research article or thesis, however gender data were converted to percentages where applicable, and age ranges were favoured where available. Ethnicities were also recorded as specified, however for consistency, terms such as “Caucasian” and “New Zealand European” were recorded as “European” for the purposes of this review, and these data were also converted to percentages where applicable. The key findings were summarised based upon information within abstracts and full texts. Lastly, each study was categorised according to the primary methodology used, while those that analysed data from existing treatment trial and survey datasets were labelled as secondary analyses.

The scoping review has been registered on OSF (https://osf.io/c8jwn). No ethical approval was required for this review.

## Results

### Total records included

**Fig. 1:**
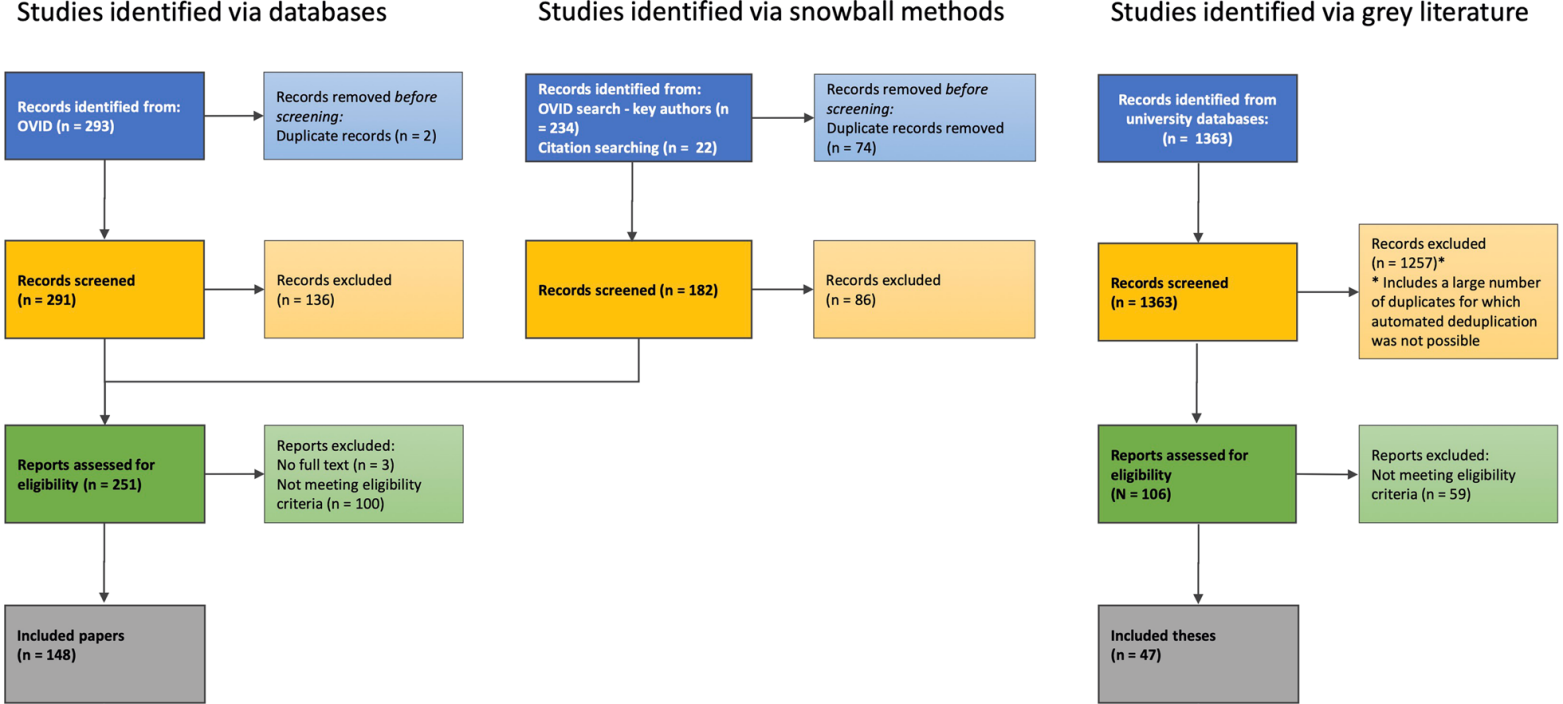
PRISMA flowchart depicting record identification process and number of records included or removed at each stage

**Fig. 2:**
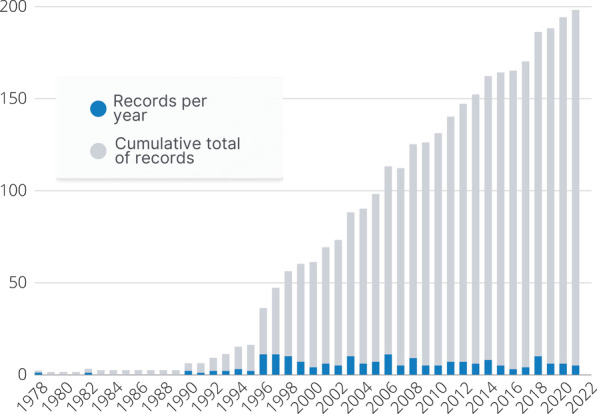
Number of included theses or journal articles published each year and cumulative totals

The total number of records identified and excluded at each step of the literature search are detailed in Fig. 1. A total of 195 records were included in the final review, with 148 journal articles and 47 theses (13 Doctoral, 34 Master’s) having met full eligibility criteria for the study. Journal articles were published between December 1978 and May 2021, while theses were completed between 1990 and 2021. The specific completion dates for two theses finalised in 2021 were unable to be verified, however the decision was made to include those in the review. The number of publications per year, in addition to the cumulative total of publications, is shown in Fig. 2.

### Study classifications

Study methodologies across the journal articles and theses fell into seven broad categories of treatment trials (18 records, Table 1), secondary analyses of existing datasets (50 records, Table 2), non-treatment experimental interventions (17 records, Table 3), cross-sectional research (63 records, Table 4), case control studies (9 records, Table 5), qualitative or mixed-methods (28 records, Table 6), or case studies and series (10 records, Table 7).

**Table 1:** Treatment trials

References	Population focus	Focus	Key data collected	Sample n	Gender	Age	Ethnicity	Summary findings
Babbott [59]*	Non-clinical (NC)	Non-concurrent multiple baseline: Trialling acceptance and commitment therapy for disordered eating	EAT-26, AAQ, SWLS, SA-45	17	12% M 88% F	18–64	64.7% European, 5.9% Māori, 11.8% Indian, 11.8% Latin American, 5.9% South African	Significant decrease in eating pathology, but not general pathology
Bulik [18]	BN (BTS)	RCT: Results from end of RCT and follow-up at 6 and 12 months. Therapies were CBT + then randomisation to 1) exposure with response prevention to binges (B-ERP), 2) to purging (P-ERP) or 3) relaxation	Physiological, biological measures, self-report measures, SCID I and II, HDRS, GAF, EDI	135	F	17–45	BTS sample 91% European 6% Maori, Pasifika, Asian	All therapies were effective and did not differ on abstinence or binge purge frequency. B-ERP had advantage for other ED symptoms, and mood but this was not maintained over follow-up
Carter [60]	BN (BTS)	RCT: 3-year follow up of BTS	Structured interview of ED symptoms, EDI, HDRS, GAF	135 (113 at follow up)	F	17–45	BTS sample	At the 3-year follow-up, 85% of the sample had no current diagnosis of bulimia nervosa. Failure to complete CBT was associated with inferior outcome. No differential effects were found for exposure versus nonexposure-based treatment
Carter [61]	AN (ATS)	RCT: long-term efficacy of three psychotherapies for AN (ATS)	SCID (DSM-IV). Global AN symptom status,, physical, cognitive and behavioural ED measures, EDE, EDI-2, GAF, HDRS	43	F	17–40	ATS sample 100% European	SSCM advantage over CBT and IPT during treatment was not sustained. All effective bus no significant differences among treatments at follow-up
Clyne [62]	BED	Single case design with multiple baseline evaluation: preliminary trial of a psychoeducational group programme of emotion regulation for treatment of BED	Daily Log of Eating and Emotions, BES, QEWP, DASS, PSS, The COPE, EIS, TAS-20, ATSS	11	F	18–69	100% European	Reduced binge-eating, alexithymia, stress, and depression. Improvements in cognition. At 2/3 month follow up, all participants no longer met criteria for BED
Clyne [63]	BED	Non-randomised with waitlist control group: regulation of negative emotion as a possible BED treatment	QEWP, EDE, EDE-Q, BES, EES	23	F	18–65	91.3% European, 4.3% Māori, 4.3% Other	Treatment outcomes comparable to existing therapies for BED
Davey [64]*	BN, AN, EDNOS, NC	Quasi-experimental (non-randomised) 2-group comparison: Efficacy of two pre-treatment interventions focused on motivation. Groups were motivation + education versus motivation alone	EDE-Q4, BDI-II, Dflex, MSOC, Change Continuum	252	97% F, 3%M	11–62	88.5% European, 4.8% Māori, 4.8% Asian, 0.8% Pasifika, 0.4% South American, 0.8% Middle Eastern	Improvements in motivational stage of change were observed in both groups, while improvements in patient readiness, confidence and importance to change as well as treatment attendance were identified in the pure Motivation Group
de Hoedt Norgrove [65]*	Emotional eaters	Multiple baseline design: Acceptance and commitment therapy (ACT) for emotional eating using a multiple baseline	Feedback questionnaire, MEAQ, valuing questionnaire, AAO, CES, GHQ, journal entries (e.g. frequency of unhealthy eating)	8	6 F 2 M	18–52	75% European, 12.5% European/Māori, 12.5% Māori/ Pasifika	Reduction in binge eating, associated with decreased experiential avoidance and cognitive inflexibility
McIntosh [17]	AN (ATS)	RCT: comparing efficacy of CBT versus IPT versus a control therapy (nonspecific supportive clinical management	Global AN symptom status, SCID for DSM-IV, EDE, HDRS, GAF, EDI-2	56	F	17–40	ATS sample 96% European	Nonspecific supportive clinical management (subsequently called SSCM) superior in completers and intention to treat analyses
McIntosh [66]	BN (BTS)	RCT: Long-term follow up of participants from RCT for BN	SCID, Structured interview of ED symptoms, EDI, HDRS, GAF	135 (109 at follow up)	F	14–45	BTS sample	Those in in SSCM group more likely to have a good outcome post-treatment, but no differences between groups at long-term (5 year) follow-up
McIntosh [19]	BED, BN (BEP)	RCT: efficacy of three therapies for binge eating: Standard CBT versus CBT augmented with schema therapy versus CBT with a focus on appetite	SCID-I and II, EDE-12, EDI-2, SCL-90-R	56	F	16–65	BEP sample	All groups improved but no significant differences between therapies
Mercier [67]*	BN	RCT: Tested intervention aiming to alter coping behaviours and cognitive processes in those with BN versus directly targeting clinical features. Wait-list control and follow-up design	General information questionnaire, DSSI-R, The Bulimia Test, Affectometer 2, BDI, RSES, STAI, TAI	24	F	19.3–41.1	Not stated	Decreased BN behaviours and cognitions following alternative intervention, little difference between intervention groups by 3 years
Roberts [68]	BN, AN	Single arm design: Efficacy and feedback on group cognitive remediation therapy	Dflex, Autism Quotient, EDE-Q, DASS-21, BMI, qualitative questionnaire	28	96% F 4% M	M 25.07 (SD 8.25)	Not stated	Intervention was effective and had positive qualitative feedback
Then [69]*	AN	Single arm design: Efficacy of metacognitive therapy modified for the treatment of A	BMI, EDE-Q, MCQ-30, TCQ	12	Not stated	M 22.17 (SD 5.17)	1 NZE, 2 Māori, 3 Samoan, 4 Cook island, 5 Tongan, 6 Niuean, 7 Chinese, 8 Indian, 9 other	Mixed results but there were reductions in patients positive beliefs about worry, depressive symptoms, worries and rumination levels following metacognitive therapy
Wallis [70]*	BED	Quasi-experimental (non-randomised intervention) with control: Teaching emotional discrimination and management in a group programme for those with BED	EDI-2, MHO, BDI, BAI, EES, COPE, GHQ	6 (BED n = 3, NC n = 3)	F	25–47	83% European, 17% Māori	EDI-2, EES, BDI, BAI, and COPE results indicated positive results following the programme
Wilksch [71]	NC (MS -T)	RCT: Trialling online programs for efficacy in reducing risk of disordered eating in an Australasian sample	EDE-Q	575	F	18–25	82.2% European, 8.8% Asian, other not stated	Media Smart Targeted program reduction in DE
Wilksch [72]	BED, BN, AN, OSFED, NC (MS—T)	RCT: Programme seeking to reduce risk of eating disorder diagnosis in NZ and Australia	EDE-Q	316 (MS-T n = 122 (baseline ED diagnosis n = 90): CT = 194 (baseline ED diagnosis n = 130))	F	M 20.8 (SD 2.26)	MS-T sample	At 12-month follow up MS-T participants were 75% less likely than controls to meet ED criteria, this finding was also significant amongst both non-treatment seekers and treatment seekers
Wilksch [73]	NC	RCT: An online 9-module eating disorder risk reduction program (Media Smart—Targeted (MS-T)) and control condition (positive body-image tips)	DASS-21, Mini International Neuropsychiatric Interview (dependence on alcohol, dependence on recreational drugs, high suicidality)	316	F	18–25	States most common is European and Asian	MS-T shows positive effect on eating disorder risk, as well as other mental health factors

*NC* non-clinical, *RCT* randomised-controlled trial, *EAT* Eating Attitudes Questionnaire, *AAQ* Acceptance and Action Questionnaire, *SWLS* Satisfaction with Life Scale, *SA-45* Symptom Assessment-45 Questionnaire, *SCID* Structured Clinical Interview for DSM Disorders, *HDRS* Hamilton Depression Rating Scale, *GAF* Global Assessment of Functioning Scale, *EDI* Eating Disorders Inventory, *EDE* Eating Disorder Examination, *BES* Binge Eating Scale, *QEWP* Questionnaire on Eating and Weight Patterns, *COPE* Coping Orientation to Problems Experienced Inventory, *EI* Emotional Intelligence, *TAS-20* Toronto Alexithymia Scale, *ATSS* Activated Thoughts in Simulated Situations, *EDE* Eating Disorders Examination interview, *EDE-Q* Eating Disorder Examination Questionnaire, *EES* Emotional Empathy Scale, *BDI* Beck Depression Inventory, *Dflex* Detail and Flexibility Questionnaire, *MSOC* Motivational Stages of Change, *MEAQ* Multidimensional Experiential Avoidance Questionnaire, *AAQ* The Acceptance and Action Questionnaire, *CES* Compulsive Eating Scale, *GHQ* General Health Questionnaire, *CSPRS-AN* Collaborative Study Psychotherapy Rating Scale—Anorexia Nervosa, *SCL-90-R* Symptom Checklist-90-Revised, *DSSI-R* Delusions-Symptoms-State Inventory-Revised, *RSES* Rosenberg Self-Esteem Scale, *STAI* State Trait Anxiety Inventory, *TAI* Test Anxiety Inventory, *DASS* Depression Anxiety and Stress Scale, *PSS* Perceived Stress Scale, *EIS* Emotional Intelligence Scale, *BMI* body mass index, *MCQ* Metacognition Questionnaire, *TCQ* Thought Control Questionnaire, *MHO* Middlesex Hospital Questionnaire, *COPE* Coping Orientation to Problems Experienced

*Identifies that the record is a thesis

**Table 2:** Secondary analyses

References	Population focus	Focus	Key data collected	Sample n	Gender	Age	Ethnicity	Summary findings
Anderson [74]	BN (BTS)	Temperament and character ratings at the beginning of CBT intervention and one year later	TCI, HDRS, B-ERP, P-ERP	135 (91 for this report)	F	17–45	BTS sample	Decreases in harm avoidance temperament and increase in self-directedness
Bourke [75]	BN (BTS)	Neuropsychological function in BN with comorbid psychological conditions	Diagnostic interviews, neuropsychological testing	41	F	17–45	BTS sample	Borderline personality disorder and MD together associated with impaired cognitive function
Bulik [76]	BN (BTS)	Examined BN sample with and without personality disorders, and self-directedness in predicting presence of personality disorders	SCID for DSM-III-R, HDRS, custom structured interview of BN symptoms, GAF	76	F	> 16	BTS sample	63% had 1or more personality disorder diagnoses, which were associated with greater depressive symptoms, laxative use, greater body dissatisfaction, worse global functioning, and lower self-directedness
Bulik [77]	BN (BTS)	Examining histories of anxiety disorders in those with BN	SCID I (DSM-III-R), age onset, Self-report ED symptoms	114	F	17–45	BTS sample	Anxiety disorders onset earlier than BN
Bulik [78]	BN (BTS)	Salivary reactivity to palatable food before, during, and after treatment	SCID (DSM-III-R), HDRS, Physiological responses	31	F	18–40	BTS sample	After treatment, salivation increased significantly (p = .002) over baseline after presentation of the same foods
Bulik [79]	BN (BTS)	Comparing onset of binge eating, dieting and BN in relation to clinical characteristics and personality traits	SCID modified, SCID II, HDRS, TCI	108	F	17–45	BTS sample	Dieting preceded binge eating in the majority of women with BN. In the minority of women where binge eating precedes dieting, markedly higher novelty seeking and lower harm avoidance are displayed
Bulik [80]	BN (BTS)	Comparing BN participants with/without comorbid alcohol dependence	SCID (DSM-III-R), HDRS, GAFS, EDI-2, TCI, BIS, Défense Style Questionnaire	114	F	17–45	BTS Sample	Women with comorbid BN and alcohol dependence have increased psychopathology, impulsivity and novelty seeking
Bulik [81]	BN, AN, MD	Comparing prevalence and ago of onset of adult and childhood anxiety disorders relative to primary diagnosis of BN, AN, MD and NC controls	Diagnostic Interview for Genetic Studies, SCID for DSM-III-R	68 (AN), 116 (BN), 56 (MD), 98 (NC)	F	AN: M 31.3, BN: 26.0, MD: M 30.6, NC: M 35.5	Not stated	Certain anxiety disorders (specific phobia, overanxious disorder) were non-specific risk factors for later affective and eating disorders, while others more specific (e.g. AN and antecedent OCD)
Bulik [82]	BN (BTS)	Predictors of successful BN treatment	SCID and SCID-II HDRS, GAFS, EDI-2, Bulimia Cognitive Distortions Scale TCI	98	F	17–45	BTS sample	Baseline symptomatology and personality factors predicted rapid and sustained treatment response
Bulik [83]	BN, AN (BTS, Christchurch Outcome of Depression Study, Sullivan et al. [84] study)	Personality traits and history of suicidal behaviour in BN, AN and MD	TCI	269 (AN 70; BN 152; MDD 59)	F	22–39	Not stated for AN or MDD sample but BN sample was part of the BTS sample	Suicide attempts are equally common in women with eating disorders and women with depression, and were associated with the temperament dimension of high persistence and the character dimensions of low self-directedness and high self-transcendence
Carter [85]	BN (BTS)	Examining changes in information processing speed following CBT	Stroop test performance, self-reported recent binge, vomiting, and other purging	98	F	17–45	BTS sample	Information processing speed not associated with change across BN treatment
Carter [86]	BN (BTS)	How performance on cue reactivity test predicted outcome of psychotherapy for BN	Clinician interview, EDI, HDRS, GAF, blood pressure, heart rate, salivation	135	F	17–45	BTS sample	Abstention during pre-treatment cue reactivity task was associated with better outcome at 6-month follow-up
Carter [87]	BN (BTS)	How CBT for BN changed cue reactivity and associations with self-report measures	Clinician interview, EDI, HDRS, GAF, blood pressure, heart rate, salivation	135	F	17–45	BTS sample	Association between favourable treatment outcome and low cue reactivity on self-report measures at posttreatment
Carter [88]	BN (BTS)	Evaluating specific hypotheses on the relationship of cue reactivity and outcome in BN women	Structured interview, EDI, HDRS, Axis V of DSM-III-R, self-report, physiological measures	135	F	17–45	BTS sample	Pre-treatment cue reactivity could not predict most effective treatment modality
Carter [89]	BN (BTS)	Whether having a child after BN treatment puts women at increased risk for ED or depression	SCID (DSM-III-R), life charts (key life events, e.g. pregnancy), menstrual + weight history, pregnancy/childbirth	135	F	17–45	BTS sample	Childbirth was not specifically associated with symptomatology following treatment for bulimia nervosa
Carter [90]	BN (BTS)	Factors related to childbirth reported at BN treatment follow-up	SCID, EDI, HDRS, BMI, GAF, BDI, SCL	125	F	17–45	BTS sample	Demographic variables and poor functioning following treatment predictive of non-conception
Carter [91]	BN (BTS)	Influence of pre-treatment weight across treatment and five-year follow-up	Pre-treatment BMI, BMI at follow-up	134	F	17–45	BTS sample	Participants who were overweight at baseline gained more weight than those in low and normal weight groups
Carter [92]	BN (BTS)	5-year follow-up of those who participated in BTS RCT for BN	SCID (DSM-III-R), EDI, HDRS, GAF, BMI	80	F	17–45 at treatment	ATS sample	Five years after treatment, approximately one half of the participants had changed substantially in weight. Patients who gained weight were more likely to have been heavier and more dissatisfied with their body
Carter [93]	BN (BTS)	Testing whether able to assess cue reactivity with a self-report questionnaire	Adapted Situational Appetite Measure (SAM)	135 (complete data for 82)	F	17–45	BTS sample	A self-report questionnaire provided useful information regarding cue reactivity among women treated for bulimia nervosa. Greater improvements in cue reactivity associated with favourable treatment outcomes
Carter [94]	BN (BTS, Christchurch Outcome of Depression Study, postpartum study [95])	Sex frequency, enjoyment, and issues in women with AN, MD, or in postpartum period	Social Adjustment Scale	76 (10 AN)	F	AN: 28.4 (SD 6.1)	Various samples	AN and MD groups more likely to have had sex in prior two weeks, but also more likely to report sexual problems, than postpartum group
Carter [96]	BN (BTS)	Relationship between weight suppression prior to treatment and treatment outcomes	BMI	132	F	17–45	BTS sample	Found that weight suppression did not predict treatment outcome but did predict weight gain
Carter [97]	AN (ATS)	Whether severity of weight suppression predicted total rate and amount of weight gain during AN recovery	BMI	56	F	17–45	BTS sample	Weight suppression was positively associated with total weight gain and rate of weight gain over treatment
Falloon [98]*	BED, BN (BEP)	Focused on how closely therapists in the BEP RCT adhered to each of three psychotherapies for binge eating	Collaborative Study Psychotherapy Rating Scale-Binge Eating (CSPRS-BE)	112 participants, 4 therapists	F	M 35.3 (SD 12.6)	67% NZ European, 17% other European, 9.8% Māori, 3.6% Asian, 2.7% other	Therapy modalities were distinguishable by raters blind to treatment
Gendall [99]	BN (BTS)	Comparing nutrient intake of women with BN regarding recommended dietary allowances, and to population sample	Food diaries	50 (BN) 468 (Population sample)	F	BN:17–45 Population: 19–44	BTS sample	Food eaten outside of binges episodes associated with low iron, calcium and zinc, and overall energy intake. Overcompensation for this during binge episodes
Gendall [100]	BN (BTS), MD (Christchurch Outcome of Depression study)	Comparison of visceral protein and haematological status between BN and depression controls	SCID, HDRS, structured interview of recent BN symptomatology Bloodwork (visceral protein and haematological status)	152 (BN) 68 (MD)	F	BN: 17–45 MD: 18–46	BTS and MD samples	BN and MD groups did not differ on visceral protein or haematological measures. Low prealbumin and albumin levels were associated with more frequent vomiting. High frequency of vomiting and alcohol abuse/dependence, may increase the risk of subclinical malnutrition
Gendall [101]	BN (BTS)	Factors association with BMI and weight change in BN, before, during, and after CBT treatment	HDRS, GAFS, EDI, physical measurements	94	F	17–45	BTS Sample	CBT is not usually accompanied by substantial weight gain
Gendall [102]	BN (BTS)	Menstrual cycle and associated factors in BN patients. How this changed across and after CBT treatment	Blood sampling, self-reported food/drink intake, BMI, SCID, GAFS, HDRS	82	F	17–45	BTS sample	Association between menstrual irregularity and indices of nutritional restriction, not reflected by energy intake or body weight
Gendall [103]	BN (BTS)	Blood lipid and glucose changes during and after CBT for BN (BTS)	Blood tests, BMI, SCID, HDRS	135	F	17–45	BTS sample	At 3-year follow up, plasma HDL-cholesterol increased and total cholesterol decreased significantly in the group as a whole
Gendall [104]	BN (BTS)	Thyroid hormone levels in women before and after CBT for BN	SCI for DSM-III-R, HDRS, BMI, blood samples (serum T4 and free T4)	107	F	17–45	BTS sample	Lower pre-treatment T4 associated with persisting ED at follow up
Gendall [105]	BN (BTS)	Childhood gastrointestinal (GI) issues and BN psychopathology	SCID, structured interview questions about childhood GI complaints	135	F	17–45	BTS sample	Individuals with childhood GI complaints and other risk factors for BN may be at greater risk of developing a more severe eating disorder at an earlier age
Gendall [106]	AN (ATS)	Factors associated with amenorrhea in AN	SCID (DSM-IV), HDRS, TCI, additional questions on eating/weight/treatment/menstrual status, food diary, physical measurements	39	F	23.3 ± 6.2	ATS sample	The use of exercise to control weight, low novelty seeking scores, and low systolic blood pressure were predictors of amenorrhea independent of body mass index
Jenkins [107]*	AN (ATS)	Whether motivation to recover is related to treatment outcome in those with anorexia nervosa	SCID for DSM-IV, Global AN status, motivation measures, including Motivational Interviewing Skills Code Version 2.0 Outcome Rating Scale	53	F	18–45	ATS sample	Higher levels of positive change talk (and lower levels of negative) did not associate with better treatment outcome. No significant difference in treatment outcome observed between participants with different positive/negative change talk ratios
Jordan [108]	AN (ATS)	Comparing history of anxiety and substance use disorders in those with AN and MDD	SCID for DSM-III-R	90 (40 AN; 58 MDD)	F	18–40	AN: 98% European MDD: 93% European	OCD elevated in AN compared to MDD sample
Jordan [109]	AN (ATS) BN (BTS)	Comparing lifetime comorbidities in participants with AN, BN, and major depressive disorder	SCID-P, SCID II, HDRS, GAF	56 (AN), 132 (BN), 100 (MD)	F	17–40	AN: 96% European, BN 91% European, MD 94% European	AN had higher OCD, AN-BP and BN elevated Cluster B personality disorders; all samples elevated Cluster C personality disorders
Jordan [110]	AN (ATS)	Assessing the constructs measured by YBC-EDS	YBC-EDS, BMI, HDRS, EDE-12, EDI-2	56	F	17–40	100% European (96% NZ European, 4% European born outside NZ)	Measured severity, YBCEDS sensitive to change following treatment
Jordan [111]	AN (ATS)	Clinical characteristics of participants who prematurely terminate treatment	SCID, SCID II, TCI-293, GAF, HDRS, EDE-12, EDI-2	56	F	17–40	Predominantly European	Lower self-transcendence scores associated with premature treatment termination
Jordan [112]	BN (BEP)	Comparing symptoms and comorbidities across BN-P, BN-NP, and BED groups	SCID for DSM-IV, MADRS, GAF, EDE, EDI-2	112	F	> 16	BEP sample	BN-NP sits between BN and BED but some distinct features
Jordan [113]	AN (ATS)	Process and other factors associated with treatment non-completion in AN	Treatment Credibility Scale, TCI, VTAS-R, VPPS, therapy alliance ratings	56	F	17–40	ATS sample	Predicted by treatment credibility, lower self-transcendence, and lower early therapy alliance
Lacey [114]	BN, AN, OSFED, EDNOS (PRIMHD)	Comparing clinical characteristics and health service use for EDs by Māori and non-Māori	National health database PRIMHD data	3,835	F	10+	7% Māori	Māori were under-represented in treatment services. Once in treatment, duration was comparable. Māori more likely to be treated for BED or EDNOS
McIntosh [115]	AN (ATS)	Relevance of BMI cut off in diagnosing AN	SCID for DSM-IV, EDE, HDRS, GAF, EDI-2, BIAQ, TFEQ, EAT, SCL-90, anthropometric and medical measures	56	F	17–40	ATS Sample	Little difference between strict versus lenient BMI groups
McIntosh [116]	AN (ATS)	Therapist adherence to three different psychotherapies in ATS RCT	CSPRS-AN	56 (AN) 3 therapists	FF therapists	AN: 17–40, not stated for therapists	ATS sample	Good adherence to therapy types, blind raters clearly distinguished therapies
McIntosh [117]	AN (ATS)	Assessing distinctiveness of three therapies and change over therapy in RCT for AN	CSPRS-AN (blind raters)	53	F	M 23.1	ATS sample	Therapies distinguishable, subscale measures higher for corresponding therapies, both SSCM and CBT sessions rated significantly higher in the middle stage of therapy
Rowe [118]	BN (BTS)	Whether poorer treatment outcome for those with comorbid borderline personality disorder (BPD) and BN compared to other personality disorders (PD) or no personality disorder	SCID-I and II for DSM-III-R, CBSI, HDRS, GAF, EDI, TCI, EDI	135	F	17–45	91% NZ European	Those with BN and BPD more impaired at pre-treatment for BN and comorbid BPD, but treatment outcome over 3 years of follow up was not poorer for this group
Rowe [119]	BN (BTS)	Impact of Avoidant personality disorder on BN treatment outcome over 3 years	SCID-I, SCID-II, CBSI, HDRS, GAF, self-report questionnaires including EDI	134	F	17–45	BTS sample	No impact on eating disorder symptoms, but worse depressive and psychosocial functioning at pre and post treatment
Rowe [120]	BN (BTS)	PD severity/number of PDs as a predictor of BN treatment outcome	SCID (DSM-III-R), CBSI, HDRS, GAF, EDI	134	F	17–45	BTS sample	More PDs did not impact outcome at 3 years
Rowe [121]	BN (BTS)	Personality dimensions as predictors of 5-year outcomes among BN women	SCID-I, SCID-II, GAF, EDI-2, TCI, personality reassessment, 12-month ED behaviours and mood disorders	134	F	17–45	BTS sample	No single personality measure predicted 5-year outcome, and so comprehensive personality assessment is desirable
Sullivan [122]	BN, AN (BTS)	Differences between those with BN with/without AN history	SCID, HDRS, GAFS, EDI-2, TCI, Defence Style Questionnaire	114	F	17–45	BTS sample	Some differences between those with and without prior AN, but not distinct groups
Sullivan [123]	BN, MD (BTS)	Comparing total serum cholesterol in women with BN versus depression versus population norms	SCID, HDRS, GAFS, structured interview to assess last 14 days ED behaviour, blood samples	126 (AN), 57 (MD)	F	17–45	BTS sample	BN women had markedly higher total cholesterol than depressed women, and population norms
Surgenor [124]	AN (ATS)	Association between sense of control and variability of AN	SCID-P (DSM-III-R (with psychotic screen), EDI, Shapiro Control Inventory, additional information on ED history including anthropometric measures, menstrual status, and chronicity	51	F	M 23.4 (SD 6.4)	ATS sample	Adverse overall sense of control (along with reliance on specific means of gaining control) associated with more severe eating disturbance. Greater use of a negative-assertive style of gaining control associated with a longer time since first diagnosis, desire for control significantly associated with menstrual status
Talwar [125]*	Community sample	Correlates of disordered eating behaviours in a community sample of women	EDI-2, Rosenberg Self-Esteem Scale, BMI	60	F	16–55	70.8% NZ European, 6.3% Māori	Dysfunctional eating attitudes and behaviours associated with higher perfectionism, lower self-esteem, and elevated body mass. Increased body dissatisfaction significantly predicted BN symptoms

*NC* non-clinical, *RCT* randomised-controlled trial, *MD* major depression, *TCI* Temperament and Character Inventory, *HDRS* Hamilton Depression Rating Scale, *B-ERP* Binge—exposure to response prevention to binges, *P-ERP* Purge—exposure with response prevention to purging, *SCID* Structured Clinical Interview for DSM, *GAF* Global Assessment of Functioning, *EDI* Eating Disorder Inventory, *BIS* Behavioural Inhibition System, *BCDS* Bulimia Cognitive Distortions Scale, *BMI* body mass index, *SAM* Situational Appetite Measure, *CSPRS-BE* Collaborative Study Psychotherapy Rating Scale—Binge Eating, *YBC-EDS* Yale Brown Cornell Eating Disorders Scale, *EDE* Eating Disorders Examination, *MADRS* Montgomery and Asperg Depression Rating Scale, *VTAS-R* Revised Vanderbilt Therapeutic Alliance Scale, *VPPS* Vanderbilt Psychotherapy Process Scale, *PRIMHD* Programme for the Integration of Mental Health Data, *BIAQ* Body Image Avoidance Questionnaire, *TFEQ* Three Factor Eating Questionnaire, *EAT* Eating Attitudes Test, *SCL* Symptom Checklist, *CSPRS-AN* Collaborative Study Psychotherapy Rating Scale—Anorexia Nervosa, *CBSI* Comprehensive Bulimia Severity Index, *SCID-P* structured clinical interview for DSM with psychotic screen

*Identifies that the record is a thesis

**Table 3:** Non-treatment experimental interventions

References	Population focus	Focus	Key data collected	Sample n	Gender	Age	Ethnicity	Summary findings
Boyce [126]	NC	Whether media body ideal exposure alters mood and weight satisfaction among restrained eaters, and whether changes in either direction encourage intake of food	RS-CD, DIS, BMI, single-item weight satisfaction scale (10-point), single item hunger scale (7-point), computer task to assess implicit mood, food intake	107	F	18–37	66% NZ European, 8% Chinese, 4% NZ European/Māori, 1% Māori, 21% other ethnicities	For restrained eaters, exposure to media images was associated with decreases in self-reported weight satisfaction and negative mood, but did not alter food intake
Boyce [127]	NC	Impact of advertent or inadvertent exposure to media or control images (four conditions) and subsequent weight satisfaction and eating among restrained eaters	RS-CD, DIS, single item weight satisfaction scale (10-point), visual analogue scale of hunger, food intake	174	F	M 20.43 (SD 6.29)	79% NZ European, 5% Chinese, 5% NZ European/Māori, 2% Indian, 9% other ethnicities	Advertent (but not inadvertent) exposure to body ideal images triggered eating by restrained eaters. Neither media exposure condition impacted their weight satisfaction
Bulik [24]	BN, NC	Whether alcohol consumption differed between food deprivation and no food deprivation conditions	Behavioural	5	F	M 25.6 ± 5.6	Not stated	More alcohol consumed in non-deprived condition
Bulik [128]	BN	Examining the reinforcing value of cigarettes and food after food deprivation in female smokers with and without BN	Behavioural	10 (4 BN)	F	18–33	Not stated	Increase in reinforcing value of food, and time spent working for cigarettes after deprivation in control but not BN women
Bulik [129]	BN, NC	Effect of coffee in BN and controls during food deprivation and no deprivation	Likert scale ratings, game responses to earn coffee	10	F	BN: 32.0 ± 6.1, NC: 21.7 ± 3.8	Not stated	Those with BN consumed more coffee in deprivation condition versus control group
Bulik [130]	BN (BTS)	Salivation at presentation of food in BN sample, restrained eaters, and unrestrained eaters	SCID for DSM-III-R	57 (19 BN)	F	BN: 27·7 ± 5·8	Part of BTS sample	BN woman displayed significantly lower salivary reactivity than restrained or unrestrained eaters
Carter [131]	BN	Examining cue reactivity methodology	SCID, self-report on urge to binge/purge, assessor evaluated urge to restrict, heart rate, blood pressure	7 (BN) 13 (Control)	F	BN: M 26, NCM 28	Not stated	Recommendations for cue reactivity assessment procedure are given, emphasising standardisation of measures, and participant-specific cues
Carter [132]	BN	Evaluated body image assessment and cue reactivity in women with BN in response to a range of cues	Silhouette method for assessing body image, BDI, EDI, self-report	7 (BN) 8 (NC)	F	18–40	Not stated	BN women rated bodies as larger, and had lower body image satisfaction versus NC women. Body satisfaction ratings were not affected by cue presentation. High-risk food cues were sufficient to elicit urges to binge in BN women
Carter [133]	BN	Information processing speed and cue reactivity in BN woman in response to cues	Stroop colour-naming tasks, BDI, DRS, EDI, Self-report measures on low mood, urge to eat/binge, confidence to resist this	13 (6 BN)	F	18–40	Not stated	Specific cue types, as well as the way they were presented affected speed of information processing suggesting a more complex relationship than was anticipated
Gendall [134]	Cravers	Effect of meal macronutrient composition on subsequent behaviour and mood	Appetite and mood ratings (60 mm VAS) pre and post-test meals	9	F	38–46	Not stated	Consumption of protein-rich meals increases susceptibility to craving sweet-tasting foods in vulnerable women
Gendall [135]	Cravers	Meal induced change in tryptophan in relation to craving and binge eating	Blood sample assays	9	F	34.9–50.4	Not stated	Reduced plasma tryp:LNAA ratio (induced via high protein meal) reduced urge to binge
Hickford [136]	NC	Comparing restrained and unrestrained eaters' cognitions	BDI, Restraint scale (short), SCID for ED modules of DSM-III-R	10	F	18–40	Not stated	No difference in frequency of food cognitions between groups
Latner [137]	BED, BN	Comparing food intake between those ingesting high-carbohydrate or high-protein supplements	EDE, BDI-II, PRIME-MD	18	F	34.78 ± 9.80	Not stated	Protein supplement led to less binge-eating
Latner [138]	BED, NC	Whether energy density of meals affects intake in BED and NC	Behavioural data, EDE, EAT, DASS, BMI	30 (15 BED, 15 NC)	F	M 27.0 (SD 8.25)	63.3% European, 10% Māori, 6.7% Pasifika, 6.7% Asian, 6.7% Indian, 6.7% other	Energy intake significantly lower in the low-ED condition than high-ED condition. BED participants report lower satiation. Decreasing energy density of food consumed may help satiation disturbances
Latner [139]*	BED, NC	Effects of two different food volumes (same total calories) on subsequent appetite and intake	Ratings (VAS, 5-point scale) for appetite and eating, food diary, food intake	30 (15 BED, 15 NC)	F	M 27.07 (SD 8.24)	Not stated	Decreases in hunger, desire to eat, and loss of control were observed following higher volume food preloads. BED participants displayed greater desire and excitement to eat than controls
Stock [140]	NC	Body image relationship with body functionality versus body control	Big Five Inventory, Iowa-Netherlands Comparison Scale (INCOM), VAS for body image measures, self-objectification questionnaire (SOQ), RSES, food choice questionnaire, VAS for mood	131	F	18–35	Not stated	No increase in body satisfaction, but and lower self-objectification over time in body functionality group. Higher neuroticism associated with lower body satisfaction. Body image group participants reported lower self-esteem
Walsh [141]*	BN, NC	Examining neuroendocrine and neuropsychological functioning in individuals with eating disorders	BDI, EAT, blood testing, subjective ratings of physical symptoms	Study 1: 15 (NC), Study 2: 12 (NC), Study 3: 20 (12 NC, 8 recovered BN)	F	19–37	Not stated	Trytophan-free amino acid drink administration did not impact mood or food intake. Moderate dieting associated with alterations in brain serotonin function in women

*NC* non-clinical, *RS-CD* Restraint Scale—Concern for dieting subscale, *DIS* Dietary Intent Scale, *BMI* body mass index, *SCID* Structured Clinical Interview for DSM, *DRS* Disability Rating Scale, *EDI* Eating Disorder Inventory, *VAS* Visual Analogue Scale, *BDI* Beck Depression Inventory, *EDE* Eating Disorder Examination, *EAT* Eating Attitudes Test, *DASS* Depression Anxiety and Stress Scale, *SOQ* Self-Objectification Questionnaire, *RSES* Rosenberg Self Esteem Scale

*Identifies that the record is a thesis

**Table 4:** Cross-sectional research

References	Population focus	Focus	Data collected	Sample n	Gender	Age	Ethnicity	Summary findings
Baxter [142]	BN, AN (TRH)	Mental health conditions among Māori participants in Te Rau Hinengaro	CIDI for DSM-IV	2595	60% F 40% M	16+	100% Māori (only Māori participants from TRH)	ED lifetime prevalence of 0.7% AN and 2.4% BN
Bensley [143]*	NC (OSSLS2)	Body image among adolescents and association with different lifestyle behaviours	Otago Students Secondary School Lifestyle Survey (OSSLS2): Subscales from the Food, Feelings, Behaviours, and Body Image Questionnaire (FFBBQ), BMI, DQI	681	56% F 44% M	15–18	74% NZ European, 9% Māori, 1% Pasifika, 7% Asian, 8% other	Females had higher scores on all subscales (figure dissatisfaction, fear of weight gain, dietary restraint, and concern about eating and weight), as did those who were overweight and obese. High levels of body dissatisfaction not limited to those who were overweight and obese)
Blackmore [144]*	NC	Explored self-induced vomiting after drinking alcohol in relation to eating disorder pathology among university students	EAT-26, MAST, Drinking Habits Questionnaire, BULIT-R, CES-D, AUDIT	261	59% F 38% M	17–35	Predominantly European	For females, alcohol-related self-induced vomiting was associated with eating disorder pathology
Boyes [145]	NC	Healthy and unhealthy dieting behaviours in university couples	Perceived Relationship Quality Components Scale, RSES, BDI-II, WCBS, additional Likert scales	114	50% F 50% M	15–57	Predominantly European	More body satisfaction among F with higher SE and lower depressive symptoms. More depressive symptoms and relationship dissatisfaction for men associated with more dieting and BD in F partners. M dieted more when F partners higher SE and fewer depressive symptoms
Brewis [146]	NC	Body image in Samoan participants living in Samoa and New Zealand	BMI, custom questionnaires	226	55% F 45% M	25–55	100% Samoan	Body dissatisfaction and slim ideals common, weight loss attempts and body perceptions not different between those above versus below BMI of 27
Bushnell (1990) [147]	Population sample (CPES)	Bulimia prevalence in Christchurch population sample, oversampled for younger women	Diagnostic Interview Schedule	1498	66% F 34% M	18–64	93% European	Widespread disordered eating behaviours/attitudes, cohort effect for younger women
Chan [148]	NC	Relationship between perfectionism and ED symptoms in Chinese immigrants, and the role of ethnic identity	EDI, PANAS, MEIM, MCSDS	301	59% F 41% M	M 22.37	100% reported Chinese ancestry	Relationship between ED symptomatology and perfectionism mediated by cultural identity. Strong sense of belonging and attachment to Chinese culture appears to be protective
Dameh [149]*	AN	Evaluating insight, as well as factors that may affect this, in participants meeting DSM-IV criteria for anorexia nervosa	Markova and Berrios Insight Scale (MBIS), SAI, EAT-26	18	F	17–43	Not stated	Impaired insight in those with AN was associated with features of illness, ED/behaviours and history of abuse
Durso [150]	NC	Testing weight bias scale and associations between self-directed weight bias and other factors	Weight Bias Internalisation Scale	198 (1 NZ participant)	Not specified for NZ	Not stated for NZ	Not stated for NZ	Scale had good internal consistency and linked to other factors related to body image and ED
Fear [151]	NC	Self-reported disordered eating/attitudes in female secondary school students	BMI, EDE-2, BMI	363	F	M 14.9 (SD 0.4)	77% European, 16% Māori, 3% Samoan, 4% other	Most students wished to be smaller size, high prevalence of ED behaviours
Foliaki [152]	Population sample	Prevalence of psychiatric disorders among Pasifika in New Zealand	CIDI	2374	52% F, 48% M	16 +	100% Pasifika	12-month prevalence 1.5%; lifetime ED prevalence 4.4%
Gendall [153]	NC	Exploring food cravings in young women within the community	DIGS, custom food craving questionnaire	101	F	18–45	98% European	History of cravings common (58%) within this sample. Narrowing definition meant that fewer (28%) met criteria. Multiple core features more common for those with strong cravings
Gendall [154]	NC	Characteristics of individuals who reported cravings for food	DIGS, TCI, EDI	101	F	23–46	Not stated	Food cravings associated with alcohol abuse/dependence and also novelty seeking, high rates of ED symptoms
Gendall [155]	AN	Food cravings and intensity of craving in those with past history of AN and NC	DIGS, TFEQ, TCI	101	F	35 ± 6	Not stated	Greater proportion of those with previous AN reported strong and more intense cravings
Gendall [156]	NC	Can aspects of restrained eating be predicted using the Temperament and Character Inventory (TCI)	DIGS, TCI, TFEQ	101	F	18–45	Not stated	Low self-directedness related to higher TFEQ score, disinhibition, and hunger susceptibility. High self-transcendence related to higher TFEQ score and cognitive restraint
Gendall [157]	NC	Comparing those who crave food and binge eat versus those who crave and do not subsequently binge	Food	223	F	18–46	Not stated	Cravers who binged tended to have higher BMI, higher frequency of diagnosed BN, elevated dietary restraint, and lower self-directedness
Gibson [42]	NC	Body image scores for rugby union players	Body composition, custom version of Low Energy Availability Amongst New Zealand Athletes, EDI-3	26	M	19–28	Not stated	High prevalence of disordered eating behaviours, disturbances in body image
Griffiths [28]	NC	Anabolic androgenic steroid use/contemplation and associations with factors including body dissatisfaction and ED symptoms in sexual minority men	Online survey: Self-report weight/height, sexuality, anabolic steroid use/consideration, MBAS-R, EDE-QS, BBQ	1797 from Aus 514 from NZ	99.1% M, 0.4% other (same sample in refs 24–26)	18–78 years	Reported as Aus NZ and Non-Aus NZ	ED symptoms and dissatisfaction with muscularity and height more prevalent among those who use AAS, while dissatisfaction with body fat less common in this group
Griffiths [27]	NC (Griffiths et al. [28]sample)	Pornography use and body image, associated behaviours, and quality of life in sexual minority men	Online survey: self-reported weight and height, sexuality, MBAS-R, EDE-QS	1797 from Aus 514 from NZ	99.1% M, 0.4% other	18–78	Not stated for NZ	Increased pornography use was weakly associated with more body dissatisfaction and thoughts of anabolic steroids use
Griffiths [29]	NC (Griffiths et al. (2017) sample) [28]	Social media use and body image, ED symptoms, and steroid use contemplation in sexual minority men	Online survey: self-reported social media/dating use, height/weight, sexuality, use/thoughts of anabolic steroids, MBAS-R, EDE-QS	1797 from Aus 514 from NZ	99.1% M, 0.4% other	18–78	Not stated for NZ	Social media use positively associated with body dissatisfaction, ED symptoms, and thoughts of anabolic steroid use. Some associations strongest for image-centric platforms
Hechler [158]	Clinicians	Assess clinicians understanding of role of physical activity in AN—and describe assessment and management strategies	EDSCS (Eating disorder specialist/clinician survey)	33	Not stated	Not stated	Reported as Aus/NZ	The majority of specialists consider physical activity to be important in EDs, however those from an Asian background considered it to be minor in comparison to other nationalities
Hickman [159]*	BN, NC	Looking at relationships and associated attachments in those with and without BN, within a sample of university students	EDI, Close Relationship Scale, TFEQ, Relationship Satisfaction Scale	123 (unclear how many with BN symptoms)	F	18–40	Not stated	More anxious attachment and dieting in participants with bulimia
Hudson [160]*	NC	Body dissatisfaction, BMI, esteem, eating attitudes	EDE, BSQ, RSES, BMI	36	F	17–55	67% NZ European, 8% Māori, 25% Other	Elevated BMI linked to higher body dissatisfaction
Jenkins [161]*	NC	Eating disorder symptomatology among females in NZ of Chinese and other ethnicities	EAT-40, Eating Disorder Belief Questionnaire, additional custom questions, Perceived Sociocultural Pressure Scale, SEED, ratings of body image figures	116	F	18–47	34% Chinese, 5% Taiwan, 49% NZ European, 8% NZ Māori, 1% Pasifika, 3% Other Ethnicities	More body image dissatisfaction and fear of weight gain in Chinese group. Similar pressure to be thin between groups
Jospe [162]	NC (SWIFT)	Whether association between weight/diet monitoring influenced eating disorder symptoms	EDE-Q, self-reports of ED behaviours	250	62% F, 68% M	< 18	176 European, 18 Māori, 7 Pasifika, 5 Asian	Self-monitoring did not increase ED symptoms
Kessler [1]	TRH (BED data not previously reported)	Assessing prevalence and correlates of binge eating disorder	Composite International Diagnostic Interview	24124 (7312 NZ)	Not specified for NZ	> 18	Not stated	Lifetime prevalence estimates of BED are higher than BN, fewer than half of lifetime BN or BED cases receive treatment
Kessler [26]	BED, BN (TRH)	Compared impairment and role attainment (e.g. employment) between BED and BN	CIDI, WHO-DAS	7312 from NZ (not included in occupation and earnings assessment)	Not specified for NZ	18–98	Not stated	Effects on role attainments similar for BN and BED. F less likely to be currently married, M less likely to be currently employed. Both more higher odds of work disability and more days of work impairment
Kokaua [163]*	BN, AN	Includes prediction of eating disorder prevalence among Cook Islanders in New Zealand	NZMHS, MHINZ	How to report?	How to report?	16+	Cook Island	Any eating disorder 1.4% 12 months prevalence (unadjusted) or 1.1% adjusted. Ethnic differences in eating disorders even after adjustment
Latner [164]	BED, BN, AN	Comparing quality of life ratings in those with subjective versus objective binge eating	EDE-Q, SF-36, BDI-II	53	F	M 26.30 (SD 8.98)	94% European, 2% Asian, 2% Māori, 2% Pasifika	Impaired quality of life for subjective binge episodes and compensatory behaviours. Also accounted for 27% of physical QoL variance
Latner [165]	NC	Associations between body checking/avoidance, quality of life (QoL) and disordered eating	BCQ, BIAQ, BMI, SF-36, EDE-Q, BDI-II	214	F	M 26.3 (SD 8.98)	86% European, 8% Asian, 52% Māori	Both body checking and avoidance associated with lower QoL and higher ED symptoms
Latner [166]	BED, BN, AN, EDNOS	QoL impairment due to features of EDs (e.g. eating concern, restraint, vomiting, excessive weight concerns)	EDE-Q, The Medical Outcomes Short-form Health Survey (SF-36), BDI-II	53 ED 212 NC	F	17–65	88% European, 7% Asian, 5% Māori	More EDE-Q features, particularly shape/weight concerns, were predictive of poorer QoL
Lau [167]*	NC (SuNDiAL)	Desire to lose weight and methods of losing weight, including unhealthy weight loss methods, among adolescents	Weight attitudes and motivations for food choice questionnaire, custom questions about body image and weight loss intentions and methods	370	66% F, 34%M	15–18	72% European, 14% Māori, 13% Asian, 2% Pasifika	High prevalence of weight loss intentions. Weight loss methods more common in females
Leydon [168]	NC	Eating habits among jockeys	EAT, food diaries, menstrual status, DEXA scan, body composition, anthropometry	20	70% F 30% M	Not stated	Not stated	Osteopenia and weight control efforts common among sample of jockeys
Linardon [169]	NC, BED, BN	Participant views of digital interventions for treatment and prevention of eating disorders	Custom questionnaires	722 (133 from Aus/NZ)	95% F 5% M	M 30.25 (SD 8.29)	77.1% European, 0.4%% African American, 8.6% Hispanic, 10.4% Asian, 0.6% Pasifika Island, 2.9% other	Pros and cons identified, cons included concerns about privacy and accuracy of data
Lucassen [170]	NC (YHS)	Comparing body size, weight, nutrition, and activities in sexual and gender minorities (SGM to controls	Custom survey re weight control behaviours, BMI	7769	56% F (incl. 312 S/GM females) 45% M (incl. 150 S/GM males)	13–18	49% European, 20% Māori, 13% Pasifika, 12% Asian, 6% other	More issues with nutrition, unhealthy weight control, and inactivity among sexual and gender minorities
Madden [7]	NC	Association between intuitive eating and BMI, and eating behaviours among less intuitive eaters	Intuitive Eating Scale, BMI (self-reported weight/height), Rapid Assessment of Physical Activity, additional selected questions of menopausal status, binge-eating, food intake, and rate of eating	2500	F	40–50	83% European, 11.4% Māori, 3.0% Pasifika, 85% Asian	Intuitive eating inversely associated with BMI. Partial mediation by binge-eating
Maguire [171]	AN	Ability to predict length of inpatient treatment Australasian clinical data	Clinical data	154	98% F	M 21.2 (SD 7.2)	Not stated	Difficulty in predicting length of stay, with only two factors (length of stay, 2–3 previous admissions) independently contributing to this
McCabe [172]	NC	Three studies comparing body image of those within five different countries and cultures (Fijian, Indo-Fijian, Tongans living Tonga, New Zealand Tongan, European Australians)	Interviews and questionnaires about eating behaviours and physical activity, perceptual distortion task	Study 1: 240; Study 2: 3000; Study 3: 300	50% F, 50% M	12–18	Study 1: 48 from each cultural group, Study 2: 600 from each cultural group, Study 3:100 from each Fijian cultural group and European Australians	Body image, eating, and physical activity influenced by socio-cultural environment
McCabe [173]	NC (Pacific OPIC Project)	Environmental influences on body change strategies within different cultural groups	Body Image and Body Change Questionnaire	4904 (461 NZ)	48% F, 52% M (NZ 62% F, 38% M)	12–18	Tongan	Differing messages across and within cultural groups
Miller [174]	NC	Body perception in relation to media consumption and societal ideals	The Sociocultural Attitudes Towards Appearance Questionnaire, FRS, Media Time Use, INCOM	181	66% F 34% M	17–30	84% European, 7% Māori, 3% Asian, 2% other	Greater discrepancy between ideal and perceived current body figures for women. Greater thin ideal internalisation for women. Awareness and internalisation of thinness norms predicted body perceptions for women but not men
Moss [175]*	AN, EDNOS	Body dissatisfaction and associated factors in adolescents with eating disorders	EDI-3, CAPS, PSPS, DASS-21	40 (13 AN, 7 EDNOS) 20 NC	F	M 15.75 (SD 1.52)	ED: 80% European 10% Māori, 10% other CT: 90% European, 10% Māori, 0% other	Higher maladaptive perfectionism and anxiety linked to BD, but didn’t interact as predictors of BD in ED group
Muir [176]*	AN, NC	Whether women with AN differed from low weight women without AN in recognising emotions	Performance on facial emotion recognition test (reaction speed and accuracy)	33	F	18–55	AN: 41.7% NZE, 8% Maori, 4 “other”. NC: 90.5% NZE, 2 British, 1 Russian	Shorter response time for AN group, no difference in accuracy measures
Mulgrew [177]	NC	Weight control behaviours and associated factors in young people	BAQ, MBAS-R, PHQ, modified WCBS, BMI, weight management questions	1082	75% F 25% M	18–30	79% NZEO	More weight control behaviours among females. Feelings of fatness a key predictor of weight control
Ngamanu [178]*	NC	Compared levels of body image dissatisfaction and eating pathology in Māori and Pakeha women, also examining whether the ethnic attachment of participants was associated with the body image	BMI, MEIM, FRS, EAT-26	100	F	18–50 +	34% Pakeha, 66% Māori	Body image dissatisfaction and eating pathology did not differ between groups. Level of ethnic attachment also did not impact body image satisfaction
Browne [179]	BN, AN (TRH)	Lifetime prevalence/risk of psychiatric disorders in the New Zealand population	Survey	12, 992	57% F 43% M	16+	20% Māori, 17% Pasifika, 63% Other (Part 1), 22% Māori, 18% Pasifika, 60% Other (long-form sample)	Any ED 1.7%CI 1.5, 2.1) LT prevalence AN 0.6 (CI 0.4,0.8): BN 1.3 (1.1,1.5): Females: 2.9 (CI 2.3,3.5); Males 0.5 (CI 0.3, 0.9)
O'Brien [6]	NC	Body image and self-esteem in physical education (PE) university students	Demographic questionnaires, self-reported BMI, BES, EAT-26, global self-esteem scale from the SDQIII	228	F	PE 18: 34 ± 0.64, Psychology 18: 46 ± 0.78, Year 3 PE 21:.0 ± 1.18, Year 3 Psychology20: 9 ± 1.06	Not stated	Year 3 PE students had lower self-esteem and more disordered eating
O'Brien [180]	NC	Psychosocial characteristics among those in a weight loss programme	Custom questions on reasons, MBSRQ, single item self-esteem scale	106	86% F 14% M	M 41.9 (SD 10.8)	Not stated	Key reasons for wanting to lose weight were mood, appearance, and health. Poorer self-image/self-esteem for those citing mood reasons
Overton [181]	Clinical	Comparing emotional experience of women with EDs to NC controls	EDI-2, YSQ-SI, DES-IV	130 (30 ED)	F	Cases: M 28.1 NC M 23.8	Not stated	Use of disordered eating behaviours to manipulate both positive and negative emotional states, should be recognised as an important maintenance factor
Reynolds [182]	Clinicians	Whether health professionals felt orthorexia should be recognised as an eating disorder	Custom online survey and qualitative text boxes	52	96% F 4% M	41.2 ± 11.9	Not stated	Most clinicians (71%) felt that orthorexia should be recognised as a distinct ED
Robertson [183]*	NC	Associations between body image, self-esteem, and peer and romantic relationships	Body Image and Body Change Questionnaire, Physical Attractiveness Scale, Body Image Behaviour Scale, Social Physique Anxiety Scale, Physical Appearance Comparison Scale, RSES, Self-Description Questionnaire III, Perceived Relationship Quality Components Scale	91	80% F, 20% M	17–69	Not stated	Positive relationship between body-image and self-esteem, and between body image and quality of romantic relationships. Positive relationship between self-esteem and relationships (peer and romantic). Body image predicted self-esteem and quality of peer-relationships Self-esteem predicted romantic relationship quality
Rodino [184]	Clinicians	Fertility specialists' knowledge and practices relating to eating disorder	Adapted online questionnaire	106	51% F, 49% M	25 +	Not stated	Knowledge around relevant symptoms of eating disorders, but uncertainty around ED detection. Many not satisfied with training in this area, or not confident in ability to recognise symptoms. Large majority indicated need for further education/guidelines
Rosewall [185]	NC	Risk factors for body dissatisfaction in girls	NZSEI, EAT-26, Stunkard Body Figure Drawings, EDI, CAPS, RSES, Sociocultural Influences on Body Image and Body Change Questionnaire (Perceived Pressure to Lose Weight subscale), PANAS, POTS	231	F	14–18	73.7% NZ European, 10.3% Māori, 5.6% Asian, 2.6% Pasifika and 3% Other	Risk factors for higher levels of body dissatisfaction were perfectionism, perceived media pressure, and low self-esteem
Rosewall [186]	NC	Exploring moderations of association between body dissatisfaction and disordered eating behaviours	NZSEI, ChEAT, Collins Body Figure Perceptions, EDI, CAPS, RSE, PANAS-C, Sociocultural Influences and Body Change Questionnaire, POTS (weight-based teasing subscale)	169	F	10–12	84.0% NZ European, 11% Māori, 6% Asian, 2% Pasifika, 1% Other	Body dissatisfaction and disordered eating association were moderated by personal (e.g. perfectionism, self-esteem) and environmental factors (e.g. teasing, perceived media pressure)
Rosewall [187]	NC	Psychopathology factors related to links between BMI and body dissatisfaction, and between body dissatisfaction and disordered eating	BMI, BSQ, BIA, BES, EAT-26, PAI	186	F	18–40	78.9% NZ European, 13.3% Asian/part Asian, 3.0% Māori, 1.2% Pasifika Island 3.6% other	Reporting lower BD (than would be predicted by BMI), and less disordered eating (than would be predicted by BD) was linked to lower levels of anxiety/depression and higher mood stability
Shephard [188]*	NC	Influence of family experiences related to food and self-compassion on the association between appearance ideals and body dissatisfaction	SATAQ (Revised—Female Version), BSQ, family Experiences Related to Food Questionnaire (FERFQ), self-compassion scale (SCS)	106	F	18–48	85.8% NZ European, 4.6% NZ European and 'another ethnicity', 3.8% Chinese, 1.9% Māori, 3.8% another ethnicity	Family food related experiences and self-compassion appear to be protective, moderating relationship between body dissatisfaction and thin ideal internalisation
Slater [189]*	NC	Energy intake, activity, and disordered eating behaviours in recreational athletes	EDI-3, LEAFQ	170	64% F 36% M	18–56	Not stated	Low energy availability (LEA) common but no risk of ED for most of those with LEA
Strang [190]*	Restrained eaters	Responses to Stroop test words about food, weight, and shape by restrained eaters versus unrestrained eaters	Stroop test, RS, STAI, BDI	55 (21 restrained eaters)	Only F after initial phase	Restrained: 24.33 (9.80), unrestrained: 21.85 (5.64)	Not stated	No group differences, but may have been due to minimal symptomatology in restrained eating group versus comparison groups
Talwar [12]	NC	Body image and body dissatisfaction among Māori and non-Māori participants	Multigroup Ethnic Identity Measure, BIA-G, BES	45	F	Māori: M 19.8 (SD 1.2), European: M 19.0 (SD 1.2)	50% Māori 50% European	Lower concern about weight among Māori. Stronger Māori ethnic identity was associated with lower weight concern
Utter [5]	NC	Identifying 'red flag' behaviours for unhealthy weight loss	Youth'07 survey	9107	46% F 56% M	13–18	Māori, European, Pasifika, Asian (% not stated in this paper)	Meal skipping and fasting are 'red flag' behaviours associated with poor mental wellbeing
Vallance [191]	NC	ED symptoms and health related quality of life	SF-36, EDE-Q, EDI-2, BSQ, BCQ, BIAQ, BDI-II, BSI	214	F	17–65	85% European, 7.5% Asian, 6.1% Māori	DE and BD linked to lower quality of life
Vaňousová [192]	NC	Evaluating validity of the Eating Concerns (EAT) scale from the MPPI-3	MPPI-3 (specifically EAT scale), EPSI, EDE-Q, EDDS, BES, BI_AAQ	396	79% F 21% M	17–51	91% European, 12% Maori, 8% Chinese, 4% Indian, 2% Pasifika (some participants more than one)	Scores from new MPPI-3 EAT scale seem promising as a screening measure for eating pathology
Wells [20]	BN, AN (TRH)	Prevalence and severity of different disorders, including eating disorders, within NZ. Oversampled for Māori and Pasifika	CIDI	Short form: 12, 992, long form: 7435	57% F, 43% M	16+	20% Māori, 17% Pasifika, 63% Other (Part 1), 22% Māori, 18% Pasifika, 60% Other (long-form sample)	Any eating disorder 1.7% lifetime prevalence, 0.5% 12-month prevalence
Wells [193]	BN, AN (TRH)	Severity and interference with life for mental health conditions among NZ sample	CIDI, Sheehan Disability Scale	Part 1: 12,992, part 2: 7435	57% F, 43% M	16+	20% Māori, 17% Pasifika, 63% Other (Part 1), 22% Māori, 18% Pasifika, 60% Other (long-form sample)	Prevalence for EDs 0.5% in last 12 months

*NC* non-clinical, *CIDI* Composite International Diagnostic Interview, *BMI* body mass index, *DQI* Diet Quality Index, *EAT* Eating Attitudes Test, *MAST* Michigan Alcohol Screening Test, *CES-D* Centre for Epidemiologic Studies Depression Scale, *AUDIT* Alcohol Use Disorders Identification Test, *RSE* Rosenberg Self-Esteem Scale, *BDI* Beck Depression Inventory, *WCBS* Weight Control Behaviours Scale, *EDI* Eating Disorder Inventory, *PANAS-C* Positive and Negative Affect Scale for Children, *MEIM* Multigroup Ethnic Identity Measure, *MCSDS* Marlowe-Crowne Social Desirability Scale, *SAI* Spontaneity Assessment Inventory, *WBIS* Weight Bias Internalisation Scale, *DIGS* Diagnostic Interview for Genetic Studies, *TCI* Temperament and Character Inventory, *TFEQ* Three Factor Eating Questionnaire, *LEANZA* Low Energy Availability Amongst New Zealand Athletes, *MBAS-R* Revised Male Body Attitudes Scale, *EDE-QS* Eating Disorder Examination Questionnaire Short, *BBQ* Brunnsviken Brief Quality of Life Scale, *EDSCS* Eating Disorder Specialist/Clinician Survey, *SDQIII* Self-Description Questionnaire III, *BSQ* Body Shape Questionnaire, *SEED* Short Evaluation of Eating Disorders, *NZMHS* World Health Organisation Disability Assessment Schedule, New Zealand Mental Health Survey, *MHINZ* Mental Health Information New Zealand, *SF-36* 36 Item Short-Form Survey, *BIAQ* Body Image Avoidance Questionnaire, *BCQ* Body Checking Questionnaire, *EDE* Eating Disorders Examination, *FRS* Figure Rating Scale, *INCOM* Iowa-Netherlands Comparison Scale, *CAPS* Clinician Administered PTSD Scale for DSM, *PSPS* Perceived Sociocultural Pressure Scale, *DASS* Depression Anxiety and Stress Scale, *BAQ* Body Attitudes Questionnaire, *PHQ* Patient Health Questionnaire, *BES* Binge Eating Scale, *MBSRQ* Multidimensional Body-Self Relations Questionnaire, *YSQ-SI* Young Schema Questionnaire—Social Isolation, *DES* Differential Emotions Scale, *PANAS* Positive and Negative Affect Scale, *CAPS* Clinician Administered PTSD Scale, *POTS* The Perception of Teasing Scale, *NZSEI* New Zealand Socioeconomic Index, *ChEAT* Children’s Version of the Eating Attitudes Test, *EDI-BD* Eating Disorders Inventory—Body Dissatisfaction scale, *BIA* Body Image Assessment, *PAI* Personality Assessment Inventory, *LEAFQ* Low Energy Availability Questionnaire, *STAI* State-Trait Anxiety Inventory, *BIA-G* Group Administered Version of the Body Image Assessment, *MMPI* Minnesota Multiphasic Personality Inventory, *EPSI* Eating Pathology Symptoms Inventory, *EDDS* The Eating Disorder Diagnostic Scale, *BI_AAQ* Body Image—Acceptance and Action Questionnaire

*Identifies that the record is a thesis

**Table 5:** Studies using case-control methodologies

References	Population focus	Focus	Key data collected	Sample n	Gender	Age	Ethnicity	Summary findings
Archer [194]*	BN, AN	Exploring factors associated with AN and BN and how these may underlie dysfunctional cognitions seen in these disorders	EDI-2, BDI, MPS, Setting Conditions for Anorexia Scale, TPQ Harm Avoidance, PBI, FES	135	F	18–44	CT: 100% European. Not stated for other groups	Dysfunctional perfectionism (e.g. MPS concern over mistakes, personal standards, and parental criticism, and TPQ harm avoidance) a key personality characteristic in AN and BN
Bulik [195]	BN	Linking perceptions of family of origin in those with BN, BN and comorbid SUD, and NC	Diagnostic Interview Schedule version III-A, Family Environment Scale self-report, Semi-structured Family Environment Interview Q-Sort	63 (33 BN)	F	Not stated	Not stated	BN with no substance use disorder: group mothers viewed as more neurotic. BN group fathers perceived as more seductive. Mother of BN with substance use disorder placed emphasis on weight/appearance/exercise
Bulik [196]	AN	Assessing predictors of BN in women with AN	Medical records, Diagnostic Interview for Genetic Studies (modified)	69	F	23–72	Not stated	Highest risk window for developing BN is within 2 years after onset of AN
Bulik [197]	AN	Examining fertility and reproductive history in women with AN versus NC group	Interviews about fertility and related history	98(66 AN)	F	AN: M 32.4 (SD 8.0), NC: M 35.5 (SD 6.2)	Not stated	More miscarriages and caesareans in AN group
Bulik [198]	AN	Assessment of relevant factors (eating attitudes, parental bonding, personality) in those with a history of AN (full recovery, partial recovery, chronically ill) and NC	EDI, TFEQ, TCI, PBI, BMI	168 (70 AN)	F	23–45	Above sample	Partially recovered and chronically ill groups reported more harm avoidance, and lower self-directedness and cooperativeness, compared with fully recovered and control groups. Lower parental care scores among chronically ill group
Fowler [199]	BED	Family factors and comorbid psychopathology in those with BED and CT with obesity	DIGS, FH-RDC, PBI, FES	40 (20 BED, 20 CT)	F	M 38.8 (SD 9.8)	Not stated	BED associated with affective and anxiety disorders, and with family history of BED but not substance misuse. BED linked to “affectionless control” parenting style in the PBI, and numerous difficulties on the FES
Latner [200[]	BN	Association of psychopathology with objective and subjective bulimic episodes	EDE, TFEQ, EDI, DASS	81	F	M 28.11	81% European 10% Asian 7% NZ Māori 2% Pasifika	Frequency of objective and subjective bulimic episodes correlated with general eating psychopathology measures, and with measures of depression, anxiety, and stress
Romans [201]	BN, AN	Experience of childhood sexual abuse (CSA) prior to developing ED	PSE, ICD-10, PBI, custom interview questions (CSA)	477	F	18+	Not stated	Higher rates of EDs among group who experienced CSA. ED risk factors among those with history of CSA were early paternal overcontrol and early puberty
Sullivan [84]	AN	Follow up of those with AN referred to eating disorders service within a 3-year period, an average of 12 years prior to the time of follow up	DIGS, GAF	168 (70 AN)	F	AN M 32.4 (SD 7.8), comparison M 35.5 (SD 6.2)	AN: 98.6% European, comparison: 96.9% European	AN group persistence in low body weight, perfectionism, and cognitive restraint

*NC* non-clinical, *CT* controls, *EDI* Eating Disorder Inventory, *BDI* Beck Depression Inventory, *MPS* Multidimensional Perfectionism Scale, *TPQ* Tridimensional Personality Questionnaire, *PBI* Parental Bonding Instrument, *FES* Family Environment Scale, *FEI* Family Environment Interview, *DIGS* Diagnostic Interview for Genetic Studies, *SCID* Structured Clinical Interview for DSM, *TFEQ* Three Factor Eating Questionnaire, *TCI* Temperament and Character Inventory, *BMI* body mass index, *FH-RDC* Family History—Research Diagnostic Criteria, *DASS* Depression Anxiety and Stress Scale, *PSE* Present State Examination, *ICD* International Classification of Diseases, *GAF* Global Assessment of Function

*Identifies that the record is a thesis

**Table 6:** Qualitative and mixed-methods studies

References	Population focus	Focus	Key data collected	Sample n	Gender	Age	Ethnicity	Summary findings
Allison [202]*	NC	Feminist approach exploring issues related to young women’s body perception and eating behaviours	Thematic analysis of journal entries	15	F	14–16	10 European, 1 Samoan, 1 South American, 1 Irish-English, 1 Chinese-European-Eurasian, 1 not stated	Identified Western cultural influences on eating behaviours and body image
Barry [202]*	NC	Issues with eating, weight, and body image in women with type 1 diabetes and health professionals	Semi-structured interviews	17 (12 with type 1 diabetes, 5 health professionals)	F	16–25	Not stated	Different perceptions of health professionals versus young women with Type 1 diabetes. Eating and weight related disturbance (including insulin omission) reported
Batenburg [203]*	AN	Experiences and opinions of those who had experienced and recovered from anorexia nervosa	Semi-structured interviews	8	F	17–27	5 NZ European, 1 Māori/European, 1 Indian, 1 Belarusian	Model of AN aetiology developed, based on categories of perceived causes of relapse
Bellingham [204]*	AN	Parental perspective on experiences of having a child with AN	Semi-structured interviews	12	50% M, 50% F	Not stated	Not stated	Identified three stages from parental accounts, termed the insidious, tenacious, and recovery stages
Carne [205]*	NC (OPIC Project)	Included examination participants' attitude toward own weight	PedsQL, AQoL, semi-structured interviews	Quantitative: 4429, qualitative: 36 (drawn from quantitative sample)	Quantitative: 48% F, 52% M, qualitative: 50% M, 50% F	13–18	Quantitative: 59% Pasifika, 20% Māori, 11% European, 10% Asian Qualitative: 33.3% Māori, 33.3% European, 33.3% Pasifika	Lower physical QOL linked to higher weight status, high QOL for those who were obese (relative to previous findings), sociocultural factors protective against internalised stigma, friendships related to perception of own weight
Chisholm [206]*	NC	Examined relationship between dieting and factors within romantic relationships in a sample of heterosexual couples	PRQC, AAQ, RSES, BDI-21, WCBS, EDI-2, WMSI, weight-loss support helpfulness, BMI, body satisfaction (Likert scale)	88	50% F, 50% M	F: M 29.43, (SD 11.87), M: M 31.61 (SD 11.87)	Not stated	More disordered eating attitudes where lower perceived partner support. Higher levels of unhealthy dieting with lower self-esteem (mediated by disordered eating attitudes). Partner support appears protective for those with low self-esteem
Conder [207]*	NC	Explored body image and how this was constructed among women with intellectual disabilities	Semi-structured qualitative interviews	25	F	21–65	88% NZE, 8% Māori, 4% Pasifika	Themes identified were 'beauty and the body', 'a fit and functional body' and 'a gendered body'
Easter [208]*	NC	Problematic behaviours among elite athletes. Includes topic of disordered eating	Semi-structured qualitative interviews	10	50% F, 50% M	Early 20s to late 40s	80% European/Pakeha, 10% Māori, 10% Other European	A number of behaviours reported, including disordered eating. Potential influences on this behaviour included comments/criticism from others, unrealistic sociocultural standards, and media influence
Gunn [209]*	BN, AN, EDNOS, self-diagnosed	Experiences of mothers who became pregnant after having recovered from an eating disorder	Qualitative interviews	10 women with past ED, 8 without	F	27–46	European	Reported healthy pregnancies among recovered women, no difficulties with infant feeding, no tendency for undue anxiety about weight gain
Hall [210]	AN	Family factors and their association with AN	Interviews with parents of those with AN	50 (AN)	F	Not stated	European	Possible aetiological factors included socioeconomic status, and family factors such as a parental history of psychiatric and medical illness
Hammond [211]*	NC, ED	Examined body image appraisals, self-esteem, body related esteem, weight locus of control, and figure ratings in groups of women: normal weight, overweight, had ED or were body builders. Qualitative study examined self-esteem and experience of teasing	RSES, BES, WLOCS, figure rating scale, silhouette rating scale, qualitative interviews	122	F	Normal weight: M 31.14 (SD 10.40), overweight: M 38.84 (SD 12.50), ED: M 27.48 (SD 10.23), body builders: M 28.81 (SD 6.31)	89% European, 3% Maori, 4% Pasifika, 3% Other	Positive description for normal and muscular, but not thin or overweight body types. Difference between groups regarding ideal figures. Self-esteem and body esteem did not correlate for body builders. ED reported feeling bigger compared to what they thought. Similar ratings for figures seen as likely to be attractive for males
Jones [43]*	NC	Body image dissatisfaction in males involved in weight training, and potential influences and impacts on wellbeing	Semi-structured interviews	12	M	18–29	83.33% NZ European, 8.33% NZE/Māori, 8.33% Cook Island/Māori/Tahitian/Scottish	Weight training exercise related to both positive and negative body image/evaluation, observed sociocultural influences on body image Behavioural indications that participants were downplaying impact of body image dissatisfaction
Kleinbichler [212]*	AN, NC	Elaborating on knowledge surrounding metacognitive processes in AN, compared with dieting and non-dieting women	BMI, DASS, EAT-26, PSWQ, PBRS, NBRS, RRQ, TCQ, MCQ-30, EDE-Q4	131	F	Non-diet: M 21.38, diet: M 23.44 (SD 8.06), AN: M 24.0 (SD 6.00)	70% NZ European, 3% Māori, 5% Chinese, 2% Indian, 11% other, 8% multi-ethnicity	Maladaptive cognitive styles among those with AN, compared with dieting and non-dieting women. Evidence supports presence of cognitive attentional syndrome in those with AN
McClintock [213]*	NC	Influences on body image dissatisfaction/disturbance, examined in three different ways	Focus group data	Study 1: 23, Study 2: 190, Study 3: 33	F	14–18	Study 1: 73.9% Pakeha, 17.4% Māori, 8.7% other minority cultures. Study 2: 74% Pakeha, 14.5% Māori, 2, 3% Pasifika, 6.9% Asian, 1.2% South African, 1.2% other minority. Study 72.7% Pakeha, 15.2% Māori, 3% Pasifika	Identified important role of social evaluation for influencing body image and unhealthy dieting behaviour, and interrelationships between sociocultural and interpersonal influences
Poulter [214]	NC	Explore perspectives of female undergraduate students with positive body image	Body image questions, BAS, BESAA, SATAQ, focus group	n = 139 for screening. N = 19 for focus analysis	F	18–30	Predominantly European	Themes included body positivity with age, mindfully engaging with media content, functional conceptualisation of the body, and role of religious and cultural identities. Women with positive body image utilise a body-protective filter, favouring body-positive information from environment
Schofield [215]*	NC	Low energy availability and associated factors (e.g. body image, nutrition) in athletes	Qualitative data, physiological data, food record	Study 1: 15, Study 2: 11	Study 1 67% F, 33% M, Study 2: 64% F, 36% M	22.8 ± 3.8	European	Highlighted complex nature of LEA, risk impacted by sociocultural environment and type of sport
Snell [216]	Clinicians	Investigating the nurse experience in an ED inpatient service	Interview	7	Not stated	30–50	Not stated	Nurses have crucial role in ED unit with unique challenges, and therapeutic relationship with these professionals can help engage clients in treatment/recovery. At times felt that this important role was invisible
Stiles [217]*	BN, AN	Assessing which eating behaviours were perceived as being normal by clinicians, dieticians, and healthy women	EDE-Q, ONE, eating behaviour, ratings of eating behaviours shown in video (Likert scales), eating style questions, qualitative interview	67	F	18–60	Not stated	Key theme was flexibility (e.g. not having strict rules). Themes also eating in response to physiological hunger, meeting nutritional needs, eating in socially acceptable manner, eating for pleasure, and regular eating)
Surgenor [218]	AN	Identify how patients view their AN with respect to self	Semi-structured interview	5	F	17–late 20s	Not stated	Patient's 'selves' have strategically different implications for therapeutic interventions. Individual therapy could be improved by establishing an authentic basis
Surgenor [219]	AN	Can treatment drop-out for AN be predicted from routine admission data collection?	BDI, EAT-26, EDI-2, RSES	213 (treatment episodes)	F	Drop out: M 22.3, regular discharge: M 21.2	Not stated	Lower BMI, AN purging subtype, and active fluid restriction make significant independent contributions to drop-out risk
Stanley [220]*	BN, AN	Risk and protective factors for those who were identified as being at-risk of negative life outcomes, and who had originally been interviewed as 12 years prior (when they were aged 11–12 years)	Semi-structured interview	9 (1 AN and BN history)	33.3% F, 66.6% M	21–22	56% Māori, 33% Pasifika, 11% Pakeha	Identified protective factors for AN participant included intrapersonal ability (e.g. self-awareness) and external supports (e.g. family). Risk factors were self-identified aberrant cognitions, physical health, adoption, and secondary schooling
Swain-Campbell [221]	BN, AN, 'other EDs'	Satisfaction with specialist eating disorders services	Custom questionnaire (structured and open-ended questions)	120	4% M	M 27	94% European	Overall high approval, but negative commentary on some aspects of treatment (e.g. being weighed, gaining weight, stopping purging as compensatory strategy)
Teevale [222]*	NC (OPIC)	Views about eating, physical activity, and body image in Pasifika Island adolescents and parents	Study 1 Questionnaire Study 2 Qualitative individual interviews	Study 14,215 Study 2 68	Study 1 52% F, 48% M Study 2 68% F, 32% M (qualitative)	Study 1 12–20 Study 2 13–17 (qualitative)	Study 1 55.4% Pasifika, 20.2% Māori, 12.3% Asian, 12.1% European, Study 2: Pasifika	Socio-environmental influences (e.g. occupational type, health education) more relevant to health behaviours than socio-cultural factors. Qualitative study: Beliefs about eating, physical activity, and body image similar between obese and healthy-weight Pasifika participants
Thabrew [223]	AN	Exploring inpatient AN treatment experience	Semi-structured interview	9	F	15–17	7 NZ European 2 Asian	Themes identified included admission benefits (safe space, support from staff), stress (e.g. being re-fed, being away from supports and regular life), control/power (e.g. compulsory treatment), being heard, and comparison with others in treatment
Tozzi [224]	AN (Sullivan et al. [84] sample)	Subjective accounts of causes of AN and recovery	DIGS, open ended questions	69	F	M 32.3 (SD 7.8)	98.6% European	Family dysfunction most commonly cited as causal, in addition to dieting/weight loss and stress. Factors contributing to recovery included relationships and maturation
Watterson [225]*	BN, AN, BED (COSTS)	Mixed methods study of factors associated with ED maintenance and recovery, and perceptions of what contributed to successful treatment and recovery	Qualitative interview, online survey based on existing surveys by BEAT charity and Butterfly Foundation	358 (quantitative), 18 of whom also participated in qualitative interviews	F	28.2 (SD 12.2)	88.7% NZ European, 6% Māori, 1.1% Pasifika, 13.2% other (includes Chinese, European, Australian, Middle Eastern, and Indian)	Multiple causal factors endorsed across EDs, most frequent were low self-esteem, perfectionism and difficulty managing negative emotions. Need for control was higher for those with AN
Waugh [25]	BN, AN	Comparing children of those with current or past AN or BN on factors such as eating behaviours, health, development, and psychometric variables	EDI, Toddler Temperament Scale, maternal report and interviews, food diaries, videoed mealtimes	20 mothers (10 cases, 10 NC controls)	F (Children: 5 M and 5 F per group)	Cases M 30.1 (SD 3.1), NC M 30.8 (SD 3.6). Children 12–48 months	Not stated	Difficulties in children of the ED group include low birth weight, difficulties with breast feeding, and non-interactive mealtimes
Webb [226]*	AN	Features of AN as indicated by those with current or past AN	Interviews available notes and documents	7	F	18–35	Not stated	Identifies issues relating to control/ self-concept, continued concerns around food/exercise, reluctance to develop sexual relationships, and concerns around relationships with others

*NC* non-clinical, *PedsQL* Pediatric Quality of Life Inventory, *AQoL* Assessment of Quality of Life, *PRQC* Perceived Relationship Quality Components, *AAQ* Acceptance and Action Questionnaire, *RSES* Rosenberg Self-Esteem Scale, *BDI* Beck Depression Inventory, *WCBS* Weight Control Behaviours Scale, *BMI* body mass index, *BES* Binge Eating Scale, WLOCS Weight Locus of Control Scale, *DASS* Depression Anxiety and Stress Scale, *EAT* Eating Attitudes Test, *PSWQ* Penn State Worry Questionnaire, *PBRS* Positive Beliefs about Rumination Scale, *NBRS* Negative Beliefs about Rumination Scale, *RRQ* Rumination and Reflection Questionnaire, *TCQ* Thought Control Questionnaire, *MCQ-30* Metacognitive Questionnaire 30, *EDE* Eating Disorder Examination, *BAS* Body Appreciation Scale, *BESAA* Body Esteem Scale for Adolescents and Adults, *SATAQ* Sociocultural Attitudes Towards Appearance Questionnaire, *ONE* Opinions on Normalised Eating, *DIGS* Diagnostic Interview for Genetic Studies, *EDI* Eating Disorders Inventory

*Identifies that the record is a thesis

**Table 7:** Case studies and case series

References	Population focus	Focus	Key data collected	Sample n	Gender	Age	Ethnicity	Summary findings
Bulik [227]	BN	BN participant who ate large quantities of bran as a method of simultaneously bingeing and purging	Case notes	1	F	27	European	Reported positive treatment outcome following CBT including exposure with response prevention
Bulik [228]	BN	Characteristics of a woman who self-induced a miscarriage through dietary restriction and exercise	SCID I and II, self-monitoring	1	F	28	Not stated	First account of intentional use of ED behaviours to cause a miscarriage. Commentary on patient’s perspective
Bulik [229]	BN, AN (ATS)	Case of participant who combined her ED symptoms with factitious presentations	Case notes, structured interview	1	F	Late 30s	Not stated	AN and BN true comorbid conditions with Munchausen's syndrome
Hall [230]	Service data BN, AN, Atypical EDs	Examined referral patterns to the eating disorder service in Wellington from 1977 to 1986	Interviews about ED history, case record review	343	96% F	15–29	Not stated	Rates of AN were stable but BN referrals increased from 6 to 44/100,000 per year
Hill [231]	AN	Case, treatment, and outcome of an elderly woman with AN	Case notes	1	F	72	Not stated	Onset following bereavement of husband, after nine ECT treatments the eating behaviour improved and depressive symptoms diminished
McKenzie [232]	AN Service data	Patterns of inpatient hospitalisation for AN patients admitted for the first time in 1980 and 1981	Clinical data	112	89% F 11% M	20.2 ± 7.5	99% European 1% Māori	Long admissions, secondary only to schizophrenia and organic conditions., with 45% readmission within 5 years
Scott [233]*	BN, AN	Own and family’s story in relation to author's experience with BN and AN	Conversations with family members	1 (AN/BN)	F (author)	N/A	N/A	Author identifies growth following experience, AN/BN identified as something which defies logic, isolated author from others
Surgenor [234]	AN	Case of attempted suicide using nasogastric feeding tube during AN treatment	Case description	1	F	33	Not stated	Advised potential precautions around those with NG who are at risk of self-harm
Surgenor [235]	Atypical EDs	Case report on atypical eating disorder in transgendered woman	ED service assessment data, EDI-2	1	Transgender	25	Fijian-Indian, European	Insight into the co-occurrence of an ED and transgenderism
Wu [236]	BN, AN (GBDS)	ED prevalence and disability-adjusted life years in different countries between 1990 and 2017	Age standard rates (prevalence), disability-adjusted life years	Not stated for NZ	Not stated for NZ	5–50 (age groups)	Not stated for NZ	High age-standardized rates of prevalence and disability and adjusted life-years of eating disorders in Australasia

Structured Clinical Interview for DSM, *EDI* eating disorder inventory

*Identifies that the record is a thesis

### Foci and wider studies

The groups examined included binge-eating disorder (BED), bulimia nervosa (BN), anorexia nervosa (AN), Eating Disorder Not Otherwise Specified (EDNOS) or Other Specified Feeding and Eating Disorders (OSFED), orthorexia, and disordered eating or body image among non-clinical (NC) groups. Many publications reported data on a range of variables from larger studies or datasets, including the Anorexia Treatment Study (ATS) [17]; Bulimia Treatment Study (BTS) [18]; the Binge Eating Psychotherapy study (BEP) [19]; Te Rau Hinengaro (TRH) [20]; The Costs of Eating Disorders in New Zealand (COSTS) study, the Survey of Nutrition, Dietary Assessment and Lifestyles (SuNDiAL), Youth Health Surveys [21], Programme for the Integration of Mental Health Data (PRIMHD), The Collaborative Psychiatric Epidemiology Surveys (CPES) [22], and the Global Burden of Disease Study (GBDS) [23].

### Sample characteristics

A wide range of sample sizes existed within the quantitative research, with the smallest sample recorded at 5 participants [24] and the largest being 12,992 participants [20]. Within the qualitative research, the sample sizes ranged from 1 to 69 participants. The majority of publications reported all-female (137 studies) or mostly female (14 studies) participant groups. A small number focused on male participants, and on sexual minority individuals. The age range of participants was large, with the lowest age being 12 months [25] and the highest being 98 years [26]. Of the 123 studies that provided age ranges for their samples, seven included children under the age of 13 years, with two focusing specifically on children. Thirty-five included participants over 45 years, though none focused specifically on this age group. A total of 133 studies reported ethnicity data or included samples for which ethnicity was previously reported; ethnicity data were unavailable for the remaining 62 studies. Two of the records within the scope of this review focused primarily on eating disorders or body image among Māori—the Indigenous New Zealand minority population.

### Types of data collected

The majority of studies used interviews or self-report measures. Data collection instruments that were commonly used to examine eating pathology included the Eating Disorder Inventory (EDI; 24 studies) [30], EDI-2 (19 studies), [31] EDI-3 (3 studies) [32], Eating Disorder Examination (EDE) [33] or the related questionnaire EDE-Q (29 studies) [34], and the Eating Attitudes Test (EAT-26 or EAT-40) [35] questionnaires (10 studies). Various versions of the Structured Clinical Interview for the Diagnostic and Statistical Manual (SCID) [36] were also used (35 studies). Other commonly identified instruments included the Beck Depression Inventory (BDI) [37] in 18 studies, Rosenberg Self Esteem Scale (RSES) [38] in 9 studies, Hamilton Depression Rating Scale (HDRS; 31 studies) [39], and the Temperament and Character Inventory (TCI) [40] in 14 studies. Among the qualitative studies, individual interviews were most common, while the use of focus groups was minimal. With the exception of physical measures such as weight and height, other physiological methods of data collection and analysis such as blood testing (8 studies), neuroimaging, genetic testing, and other biological assessments were less common.

## Discussion

This scoping review identified studies that examined disordered eating and body image in clinical and non-clinical samples from New Zealand, and outlined the methodologies and results reported for each study. A large number of records were located and assessed, and these involved a wide range of methodologies and vastly different foci highlighting considerable progress in understanding disordered eating and body image within New Zealand.

*Methodology* Most of the literature identified in this review described quantitative research, however a smaller number of exploratory qualitative studies and case studies were also identified, with the majority being identified during grey literature searches. Longitudinal studies and follow up studies of eating disorder treatments, particularly those of five years or more, were also uncommon, which may be attributable to the high cost and attrition rates associated with this type of research. Studies included participants from both clinical samples and non-clinical samples; however, large clinical samples were uncommon, which is likely underpinned by limited funding for larger studies (given that New Zealand allocates a much smaller portion of its GDP to funding research, relative to other countries) [41]. In addition, the relatively small New Zealand population makes it difficult to recruit large samples of individuals with eating disorders, which are relatively low prevalence conditions. Self-report and interview measures were identified as being most frequently used, whereas the analysis of biological data such as blood samples, which can be helpful in understanding the impact of disordered eating, was uncommon. This may be attributable to the relative ease and affordability of survey and interview data, whereas other methods tend to require more financial and research infrastructure, resources, and expertise.

*Sex and gender* Although some of the studies included males or gender minorities, most focussed on samples that were predominantly or exclusively female. The identification of only two all-male samples [42, 43] is consistent with reports that less than 1% of all published eating disorder research focused specifically on males with these disorders [44, 45]. Several of the identified New Zealand studies of eating disorders excluded potential male participants, or excluded data provided by male survey respondents. This may be partly because the prevalence of these disorders, with the exception of BED, tends to be lower among males [46], leading to low recruitment numbers that generally preclude statistical analyses. The inclusion of male participants also necessitates adapting treatment packages or prevention strategies for these individuals, which provides further logistical challenges for researchers [47]. Although females may be an easier group to recruit from, differences in the presentation of eating disorders and body image concerns in males need to be examined further [48]. In addition, the consistently low recruitment of male participants perpetuates the notion that eating disorders primarily afflict females, while reducing the likelihood that men will come forward to participate in future research on eating disorders, or to seek treatment. There is also evidence to suggest differences in body image concerns, as well as eating disorder risk factors and presentation, among sexual minority and LGBTQIA + individuals [28]; however, very few of the identified studies explored these differences. As such, there is a need for context-specific information to assist healthcare providers in furthering their knowledge of the presentation and treatment options for men, gender minority, and LGBTQIA + individuals in New Zealand.

*Age* There was a tendency for studies to recruit adolescents and younger adults. This may be partly attributable to convenience, with university aged students being the most readily available population for non-clinical studies, while the higher prevalence of eating disorders among young people can make other age groups more difficult to sample from. We identified very few studies that included participants under the age of 13, which is of particular concern given reports that eating disorders are being increasingly identified among children [49]. Conversely, there were also fewer studies involving middle-aged or older participants, despite middle-age being associated with increased eating disorder risk for women in particular, in part related to the menopause transition [50, 51]. With increased knowledge surrounding the risk and development of eating and body image issues across different age groups in New Zealand, more targeted and effective prevention and treatment strategies may be established.

*Ancestry* Many studies did not report ethnicity data, and Māori and Pasifika peoples were typically under-represented where these data were available. The lack of Māori and Pasifika representation and inclusion marginalises these groups further, while the extent and ways they are impacted by eating disorders, disordered eating, and body image concerns remain unclear. A lack of research into eating disorders within Indigenous and minority ethnicity populations is common within international literature, which limits our understanding of how to best understand, detect, and approach the treatment of eating disorders among these groups [52]. The results of this review suggest that New Zealand is no exception to this pattern, despite the prevalence of anorexia nervosa and bulimia nervosa in Māori being similar to or higher than in the general population [53]. Food and rituals surrounding food are central to Māori and Pasifika cultures, and are important to consider when assessing and treating eating disorders in Māori and Pasifika participants [13]. It is important to assess all eating disorders in future studies, given subthreshold eating disorders and disordered eating have been found to be highly prevalent in Indigenous peoples in Australia, suggesting current diagnostic criteria may not adequately capture eating problems in underrepresented minority identity groups [54]. Therefore, future studies of eating disorders and related issues within New Zealand need to actively seek participation from Māori and Pasifika people, and explore these issues from a culturally inclusive viewpoint.

*Strengths and limitations* This review has a number of strengths. Firstly, it captures research spanning a 43-year timeframe, allowing for a thorough investigation into the nature of research on disordered eating and body image within New Zealand. Furthermore, the review has included not only peer-reviewed journal articles, but also grey literature in the form of Masters and Doctoral theses. The addition of postgraduate research has allowed for a pragmatic and inclusive examination of the work conducted using New Zealand based samples, whereas a traditional style of review may exclude valuable data present in grey literature. The present review also has several limitations, with one being that a portion of the relevant grey literature, was unavailable for screening. Some of these theses could have added to the breadth of research methodologies, participants, and foci reported in the review. Although all Medline records are indexed in Embase, it may have been beneficial to also include Medline in the search strategy, as the indexing is unique to each of these databases. In addition, although every attempt was made to pre-define which topics would be included or excluded in the search, there is still a chance of reviewer bias in choosing whether to include research that fit less clearly within the margins of the scope. This is a risk particularly with the inclusion of research on body image. For example, other reviewers might have included studies with questionnaire items that alluded to body image, e.g. “how I look” without specifying weight and shape. However, the involvement of two independent reviewers reduced the risk of bias, as any inconsistencies in the inclusion of records were carefully addressed.

*Recommendations* Given the data presented in this review, a number of recommendations have been formulated for New Zealand research in the area of eating disorders, disordered eating, and body image. Firstly, although studies of a short term and non-experimental nature are less time-consuming and cheaper, the relapsing nature of eating disorders indicates that more longitudinal studies and long-term psychotherapy follow-ups would be valuable. Future research will also benefit from utilising different assessment methods to better understand the mechanisms underlying eating disorders. These may include physiological methods such as neuroimaging, or other biometric or biological, and genomic and other—omic approaches [55–57]. This in turn would allow for a more complete physiological picture of eating disorders in New Zealand, and would aid local research in keeping pace with international research methods. A second recommendation is to include more studies of body image and eating behaviours among males and LGBTQIA + communities. As mentioned earlier, this would further contribute to an understanding of how to responsibly and appropriately approach eating disorders in these groups. Future research should also examine eating disorders and body image concerns before adolescence, and beyond the age of 45, to better address the needs of individuals affected at different life stages. Finally, the paucity of research using a representative proportion of Māori and Pasifika participants was of particular concern. Although it may be more difficult to recruit participants from ethnic minority groups, it is vitally important that researchers make every effort to do so. This should involve engaging these communities from the outset, rather than only studying them as research participants [58].

Funders should be aware of considerable need for eating disorders research to be able to better serve ill individuals and their families in New Zealand. Proposal requirements should require inclusion of men and minoritized gender and ethnic groups, even specifying a minimum percentage of males and individuals from minority ethnicity groups. Funding should be allocated and timed in a way that supports recruitment from more difficult to reach groups, such as providing budgets specifically for targeted advertising and allowing more time to focus on engaging with these participant communities. In addition, funded research should be encouraged to include these groups as active researchers, building capacity in these communities and enabling them to provide guidance throughout the study. Lastly, budgets should be sufficient to support controlled treatment trials, particularly for groups that have been understudied, and research involving techniques and methods that are novel or underutilised.

*Conclusions* This scoping review is the first comprehensive examination of research into disordered eating and body image conducted in New Zealand. By summarising the foci, methods, and results for each of these studies, the review has also highlighted many gaps and areas where further funding and research is needed, including more treatment trials and longitudinal research, more advanced methods of data collection and analysis, and the inclusion of more diverse sample groups. While it may be more difficult to recruit individuals from minority groups, the greater social connectivity provided by the internet may assist researchers in recruiting, surveying, or interviewing such groups with less difficulty than previously. This study has identified a considerable body of research, and provides important information to assist funders and researchers in benchmarking findings against samples from New Zealand.

## Data Availability

All data generated during this study are included in this published article and were extracted from existing publications.
